# Historical loss weakens competitive behavior by remodeling ventral hippocampal dynamics

**DOI:** 10.1038/s41421-024-00751-3

**Published:** 2025-02-25

**Authors:** Chuan Lai, Kai Chen, He-Zhou Huang, Xian Huang, Juan Zhang, Yu-Bo Wang, Zhiye Chen, Feng Hu, Ziyuan Guo, Heng-Ye Man, Hui-Yun Du, You-Ming Lu, Kai Shu, Dan Liu, Ling-Qiang Zhu

**Affiliations:** 1 Department of Pathophysiology, Key Lab of Neurological Disorder of Education Ministry, School of Basic Medicine, Tongji Medical College, Huazhong University of Science and Technology, Wuhan, Hubei China; 2 Department of Dermatology, Wuhan No. 1 Hospital, Tongji Medical College, Huazhong University of Science and Technology, Wuhan, Hubei China; 3 Department of Neurosurgery, Tongji Hospital, Tongji Medical College, Huazhong University of Science and Technology, Wuhan, Hubei China; 4 Center for Stem Cell and Organoid Medicine (CuSTOM), Division of Developmental Biology, Cincinnati Children’s Hospital Medical Center, Cincinnati, OH USA; 5 Department of Biology, Boston University, Boston, MA USA

**Keywords:** Mechanisms of disease, Stress signalling

## Abstract

Competitive interactions are pervasive within biological populations, where individuals engage in fierce disputes over vital resources for survival. Before the establishment of a social hierarchy within the population, this competition becomes even more intense. Historical experiences of competition significantly influence the competitive performance; individuals with a history of persistent loss are less likely to initiate attacks or win escalated contests. However, it remains unclear how historical loss directly affects the evolution of mental processes during competition and alters responses to ongoing competitive events. Here, we utilized a naturalistic food competition paradigm to track the competitive patterns of mutually unfamiliar competitors and found that a history of loss leads to reduced competitive performance. By tracking the activity of ventral hippocampal neuron ensembles, we identified clusters of neurons that responded differently to behavioral events during the competition, with their reactivity modulated by previous losses. Using a Recurrent Switch Linear Dynamical System (rSLDS), we revealed rotational dynamics in the ventral hippocampus (vHPC) during food competition, where different discrete internal states corresponded to different behavioral strategies. Moreover, historical loss modulates competitive behavior by remodeling the characteristic attributes of this rotational dynamic system. Finally, we found that an evolutionarily conserved glutamate receptor-associated protein, glutamate receptor-associated protein 1 (Grina), plays an important role in this process. By continuously monitoring the association between the attributes of the dynamic system and competitiveness, we found that restoring *Grina* expression effectively reversed the impact of historical loss on competitive performance. Together, our study reveals the rotational dynamics in the ventral hippocampus during competition and elucidates the underlying mechanisms through which historical loss shapes these processes.

## Introduction

Social competition is essential in determining individuals’ social status and has a profound influence on the mental state of humans and animals^1–3^. Previous social experiences significantly influence individuals’ behavior during social competition. For instance, a history of winning reinforces social hierarchies and increases effortful behaviors in tube test in mice^4^. In contrast, distressful social experiences reduce the likelihood of initiating attacks or winning escalated contests^5^. In *Drosophila*, prior adverse experiences weaken social competitiveness, lowering the likelihood of success in future fights and reducing territorial behaviors, threat displays, and courtship behaviors^6^. Epidemiological and clinical studies in humans also revealed that the stress from income inequalities and economic crises impairs social functioning^7,8^. In general, individuals with defeated social experiences exhibit reduced social competitiveness, which often precedes behavioral changes associated with psychiatric disorder.

In biological populations where social hierarchies are already established, the disparity in social rank between competitors plays a pivotal role in determining the outcome of this competitive interaction^9^, with the medial prefrontal cortex (mPFC) playing a significant role^4,10^. A history of winning influences social hierarchy by altering the mediodorsal thalamus (MDT) projections to the prefrontal cortex^4^. However, before hierarchies are established, the absence of structured rules leads to more frequent and intense social competition, with outcomes driven by complex factors. Whether these findings can be extended to situations without established social hierarchies remains unclear.

Additionally, current models of social competition tend to describe it in binary terms — win or lose — offering limited insights into the dynamic psychological processes individuals experience during competitive interactions in the absence of hierarchical structures. When faced with stronger opponents with little chance to win, individuals often resist rather than concede defeat outright^10^. In this context, the mental processes of the weaker individuals are not static; instead, they undergo continuous change. Neural encoding in the prefrontal cortex can predict which individual ultimately prevails^10^, even before the competition begins^11^. Furthermore, the relative rank of competitor can be decoded by the mPFC; when facing higher-ranking opponents, subordinate mice, regardless of winning or losing, show neural trajectories that occupy the same subspace^12^. This suggests that the mPFC represents advanced functions related to identity awareness rather than strategy shifts during competition. The neural mechanisms driving dynamic behavioral strategy changes, such as shifting from struggle to surrender, remain unclear.

In this study, by using a naturalistic food competition paradigm, we found that a history of loss weakens individuals’ competitive performance when facing unfamiliar opponents in the absence of established hierarchies. We focused on the vHPC as a key brain region through c-fos staining. Electrophysiological recordings revealed that historical loss reduces the excitatory synaptic efficacy of ventral hippocampus neurons and alters the response patterns of vHPC neurons to competitive behaviors. Using the rSLDS model to analyze population activity in the vHPC, we found that the neural activity of vHPC exhibited rotational dynamics during the competition process, with different discrete states corresponding to distinct behavioral strategies of the individual. Historical loss impairs competitiveness by disturbing the transitions between discrete states associated with proactive behavioral strategies. Furthermore, we identified that the evolutionarily conserved glutamate receptor-associated protein, glutamate receptor-associated protein 1 (Grina), plays an important role in this process. Conditional knockout (KO) of *Grina* in mice led to an innate deficiency in competitive ability, with notable changes in neuronal synchrony and synaptic function in the vHPC. Using the CreERT system, we restored *Grina* expression in real-time within the vHPC, which altered the dynamic velocity of the rotational dynamics system and gradually restored the competitive performance of historical loss (HL) mice. The restoration of *Grina* effectively rescued the detrimental effects of historical loss.

## Results

### Historical loss lowers down the competitive performance

**Fig. 1:**
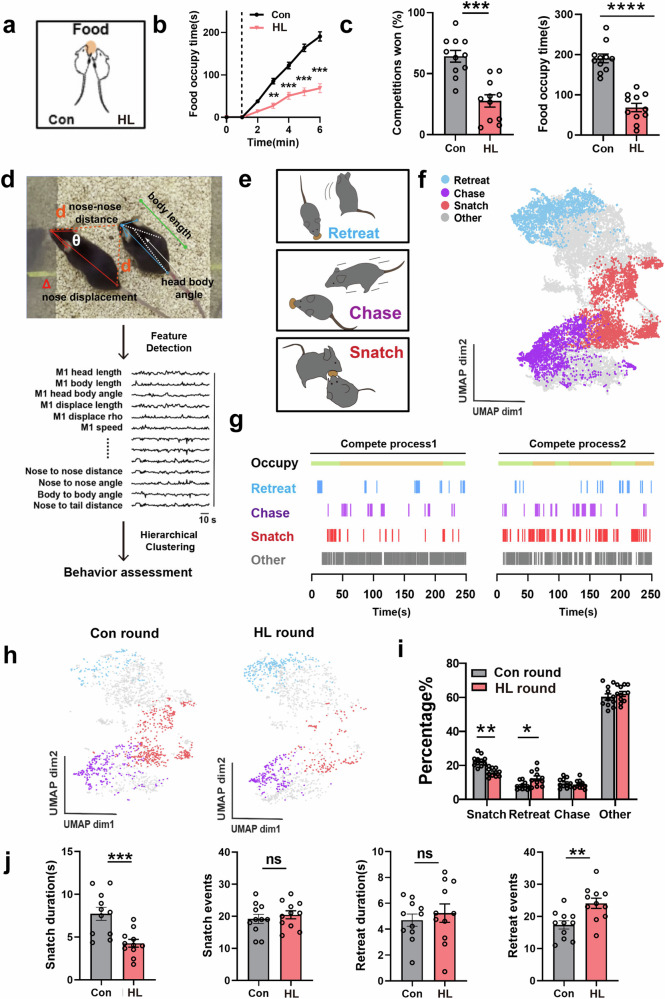
Historical loss weakens competitive performance of mice during food competition. **a** Schematic diagram of food competition. Two food-restricted mice (1 control mice and 1 HL mouse) compete for an indivisible food pellet in home cage. **b** The cumulative time that the mice occupied the food pellet during the food competition trial (**P < 0.01 and ***P < 0.001, *n* = 11 for each group). **c** Left, the winning rate of mice in each bout of scramble during competition task (*P* < 0.001, *n* = 11 for each group). Right, total food occupancy time of mice in the food competition task (*P* < 0.0001, *n* = 11 for each group). **d** The postural features extracted from video frames for subsequent behavior assessment. **e** Food competition behavioral labels by behavior classification. **f** UMAP plot showing all video clips color-coded by behavioral classification of food competition behaviors from 41 videos (32,216 points). **g** Example trials with defined behavioral labels. Fewer identity swaps of defender correspond to fewer snatch behaviors. **h** The UMAP plot of video clips color-coded by behavioral classification in Con round (Con mice as challengers) and HL round (HL mice as challengers) during a representative food competition trial. **i** Percentage of frames that correspond to specific behavioral class out of the total frames in Con and HL round, *n* = 11 mice for each group (*P < 0.05 and **P < 0.01, *n* = 11 for each group). **j** The duration and frequency of specific behavioral class in Con and HL round. Snatch duration, *P* = 0.0009; Snatch events, *P* = 0.5092; Retreat duration, *P* = 0.5353; Retreat events, *P* = 0.0037; *n* = 11 mice.

Competition for food resources is widespread in the natural world. Therefore, we designed a natural paradigm in which a pair of hungry mice compete for an inseparable food pellet within cage. After the mice were restricted in their food intake for two days to reach 80% of their body weight, they were placed in cage to compete for a food pellet (Fig. 1a; Supplementary Video S1). We divided the mice into two groups: the control group (Con), which underwent daily solo tube traversal, and the HL group, which had experienced a period of continuous loss from syn2b mice, a transgenic mouse with dominant behavior^13^ (Supplementary Fig. S1a). The HL mice indeed exhibited highly stable submissive behaviors, both in tube test and warm spot, exhibiting low competitiveness (Supplementary Fig. S1b, c). However, they did not exhibit depression or anxiety (Supplementary Fig. S1d, e), which could be attributed to the fact that this was not “Forced loss” but “Natural loss”^14^.

We paired Con mice with HL mice for food competition tests, using a round-robin pattern where each HL mouse encountered a Con mouse. Before the food competition, both were housed separately to ensure that no hierarchy had yet formed between them. We found that HL mice demonstrated lower cumulative occupancy time of the food pellet and lower success rate for food acquisition in the food competition task (Fig. 1b, c). Meanwhile, the occupancy time of the food pellet during the food competition task was positively correlated with the number of wins in the tube test (Supplementary Fig. S1f), indicating that performance in the food competition task can reflect the mice’s social competition ability. During food competition task, we defined the mouse that occupies the food pellet as the defender, and the mouse that is seeking the food pellet as the challenger. When Con mice act as challengers (Con round), they tend to approach the defender head-on and snatch the food pellet directly. However, when HL mice act as challengers (HL round), they tend to linger within a certain range of the defender for a period of time instead of immediately launching a direct competition. As shown in the relative spatial heatmap, the relative distances in the Con round are centered around null point, while a broader dispersion was observed in the HL round (Supplementary Fig. S1g). Consistent with the distribution of relative distance and angle, Con mice displayed a more concentrated distribution of unbiased relative angles and closer relative distances while HL mice exhibited a broader distribution of biased relative angles and greater distances, suggesting that Con mice preferred to choose facing the challenger directly but HL mice did not (Supplementary Fig. S1h).

Competing for food between two individuals is a complex social process, and the measurement of the food cumulative occupancy time does not adequately describe the entire competitive behavior process. To describe the behavior process during the competing processes, we employed the social LEAP estimates animal poses (SLEAP)^15^ to track various body bone nodes of the two mice in the food competition trials (Supplementary Fig. S2a). We found that after learning a sufficient amount of data, its accuracy surpassed that of human evaluation (Supplementary Fig. S2b, c). According to the spatial coordinates of the body bone nodes, we defined 26 features to describe the posture of individual mice and the relationship between competitive mice (Fig. 1d; Materials and Methods). Then, uniform manifold approximation and projection (UMAP) dimensionality reduction and unsupervised hierarchical clustering were used to divide the behaviors of mice into 18 clusters (Supplementary Fig. S2d, e). The embedding process preserves the structure of the original feature space, such as the distances between animals (Supplementary Figs. S2f, S3d). The behavioral description in UMAP clustering can be derived from the average output (Supplementary Fig. S2g) and the distribution of the original features (Supplementary Fig. S2h) in the cluster. For instance, cluster 17 exhibits a low level in nose-to-nose distance and a high head-body angle level, corresponding to a typical snatch behavior by sideways posture. Cluster 2 exhibits high levels of body length, nose-to-nose distance, angle, and the high-speed movement as average output shows, corresponding to a typical retreat behavior. Clusters 1, 6, and 8 were considered noise and were subsequently removed.

To better understand how the different behavioral clusters represent competitive processes, we divided these clusters into four types of behavior: snatch, chase, retreat, and others (Fig. 1e, f; Supplementary Fig. S2g). During the period of ownership changes for food pellets, vehement snatch behavior usually erupts and ends with the retreat of one side (Fig. 1g). We found that, for individuals as a challenger, the proportion of time spent on snatch and chase behavior is strongly positively correlated while the proportion of time spent on retreat behavior is negatively correlated with the total food pellet occupancy time, respectively (Supplementary Fig. S3a). After confirming the reliability of the behavior analysis above, we then attempted to understand the alteration of behavioral characteristics in the mice that had historical failure experiences. We categorized the situation as follows: HL round with HL mice as challengers, and Con round with control mice as challengers during the food competition. Compared to control mice, HL mice showed fewer snatch behaviors, more retreat behaviors, and comparable chase or other behaviors when they acted as challengers (Fig. 1h, i). We also focused on the differences in the original behavioral clusters between the Con and HL rounds. We observed significant differences in clusters 17 and 18, which make up the snatch behavior; while cluster 4, one component of retreat behavior, was the primary source of differences. Interestingly, cluster 16, associated with other behaviors, also showed differences (Supplementary Fig. S3b). Therefore, we conducted further analysis on cluster 16 and found that the average skeleton of this cluster exhibited a posture indicative of exploring the cage walls, characterized by a high nose-to-nose distance and increased body length. Additionally, we observed that its proportion of overall behavior had no correlation with cumulative food pellet occupancy time (Supplementary Fig. S3c). Consequently, we concluded that it is unrelated to competitive behavior.

We further compared the frequency and the duration of specific behavioral events as above in the social competition between the Con and HL rounds. Interestingly, HL mice as the challenger exhibited shorter snatch durations but not reduced snatch frequencies, as well as more retreat events but with no difference in the duration of retreats (Fig. 1j). These data indicate that historical experience of loss leads to a lower competitive performance when facing stranger opponents, manifested as shorter confrontation duration and more frequent retreat behaviors.

### Historical loss remodels the response pattern of vHPC neurons

Previous research has indicated a pivotal role of the mPFC in social competition. The relative ranks of individuals are adaptively encoded by neurons in the mPFC; when faced with opponents of varying ranks in competition, neural trajectories formed by prefrontal cortex neuronal population activity reside in distinct low-dimensional common PC subspaces^12^. However, the characteristics of the dynamics in mPFC suggest that the mPFC acts as a binary determinant encoding victory or defeat outcomes^11^ rather than the competition process itself. Therefore, we turned our attention to the upstream and downstream brain regions associated with the mPFC, with a particular focus on the ventral hippocampus (vHPC) and basolateral amygdala (BLA). These regions, via interconnecting with the prefrontal cortex, constitute a complex network ideal for the execution of the dynamic processing. Therefore, we examined whether these brain regions were activated in food competition by examining c-fos immunoreactivity. We observed significantly fewer c-fos*-*positive neurons in the vHPC but not in the mPFC or BLA of the HL mice (Supplementary Fig. S4a, b), suggesting that the ventral hippocampus’s neuronal activity change may contribute to the reduced competitive performance in HL mice.

We examined the basic synaptic properties in ventral hippocampal neurons using whole-cell patch-clamp recording (Supplementary Fig. S4c). We found that the amplitudes, but not the frequencies, of the miniature excitatory postsynaptic currents (mEPSCs) were significantly reduced in HL mice compared to control mice (Supplementary Fig. S4d–g). Moreover, a decrease in the ratio of AMPA/NMDA receptor-mediated currents was found in HL mice (Supplementary Fig. S4h, i), indicating a reduced excitatory synaptic efficacy of ventral hippocampus neurons in the HL mice.

**Fig. 2:**
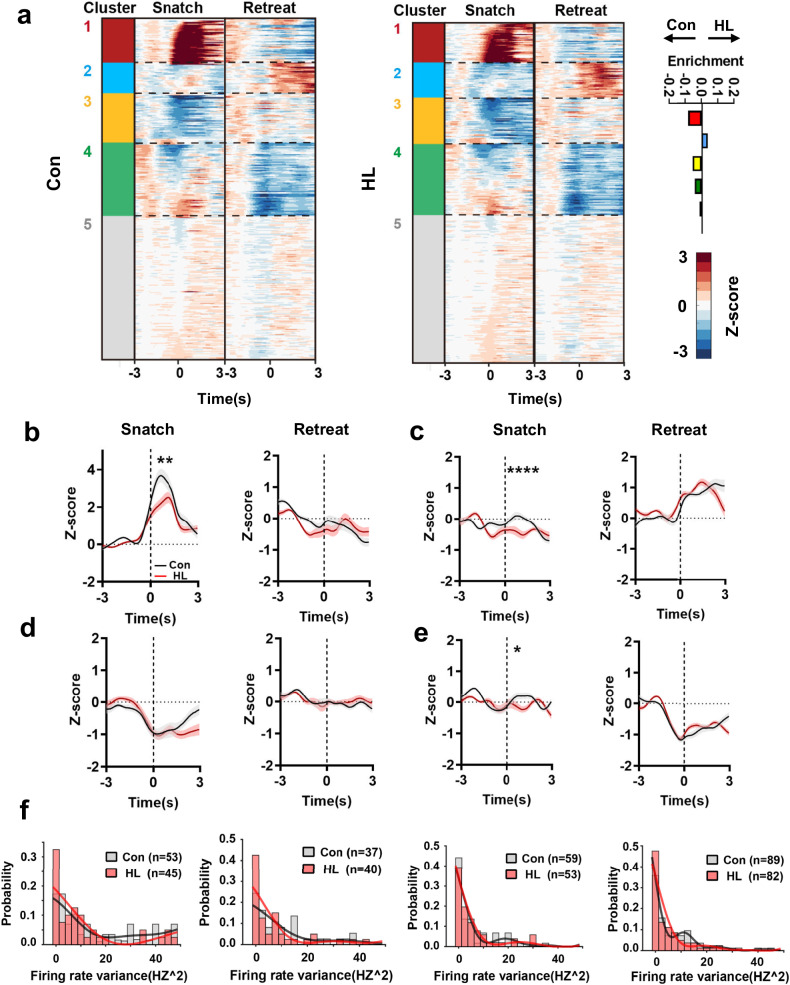
Historical loss alters the reactivity of vHPC neurons. **a** Left, heatmap of vHPC neurons responses to two specific behaviors in food competition. Colors represent clusters derived from hierarchical clustering. Cell clusters with a z-score greater than 1.5 or less than –1 were considered responsive to the event (Con, *n* = 238; HL, *n* = 220). Clusters without response are labeled in grey. Right, difference between Con and HL cells (percentage enrichment) across functional clusters. **b**–**e** Response magnitude for different cell clusters of vHPC neurons to snatch/retreat behavior events; the dotted line represents the onset of the behavior event. Cluster 1 (**b**), *n*(Con) = 53, *n*(HL) = 45, snatch *P* = 0.0068, retreat *P* = 0.0956; Cluster 2 (**c**), *n*(Con) = 37, *n*(HL) = 40, snatch *P* < 0.0001, retreat *P* = 0.0899; Cluster 3 (**d**), *n*(Con) = 59, *n*(HL) = 53, snatch *P* = 0.4653, retreat *P* = 0.5343; Cluster 4 (**e**), *n*(Con) = 89, *n*(HL) = 82, snatch *P* = 0.0368, retreat *P* = 0.4578; Wilcoxon rank sum test. **f** Distribution of firing rate variance for each neuronal cluster in Con or HL mice. The line represents the density curve of kernel density estimation using a Gaussian kernel. Black solid line, Con group; Red solid line, HL group. Cluster 1, *P* = 0.2640; Cluster 2, *P* = 0.3291; Cluster 3, *P* = 0.8186; Cluster 4, *P* = 0.9780; Kolmogorov–Smirnov test.

The most straightforward approach to determine whether ventral hippocampal neurons engage the competition process is to examine whether the response patterns of these neurons differ when mice choose different behaviors. Therefore, we evaluated the neural activity of the vHPC neurons in mice during food competition by in vivo electrophysiological recordings. The recordings revealed distinct response patterns of vHPC neurons during food competition. We aligned the neural responses with the behavioral performance of the mice during food competition and computed the mean activity of each neuron. We found that a portion of vHPC neurons were active only during specific behavioral events, such as snatch or retreat (Fig. 2a). The chase behavior was not analyzed because it typically relies on the actions of the opponent. Based on the responses to the events, we divided the vHPC neurons into 5 clusters by using unsupervised clustering (Fig. 2a). We found that different functional groups of neurons displayed unique responses to behaviors. For instance, Cluster 1 showed highly specific response to snatch event, while Cluster 2 showed highly specific response to retreat event. We next investigated whether HL treatment could affect certain vHPC ensembles at the single-cell level. Compared to the Con group, during the snatch event, the HL treatment led to a reduction in response magnitudes in neuron clusters 1, 2, and 4, whereas this effect was not observed in all clusters during the retreat events (Fig. 2b–e). Meanwhile, no significant differences were observed in the firing-rate variances between the Con and HL groups, indicating that the neuronal variability remained unchanged in both groups (Fig. 2f).

Next, we explore how these neuronal clusters change during the process of social loss. We monitored the behavior and electrophysiology of Con mice and HL group mice during social defeat bi-daily. We found that, compared to Con mice, the HL mice exhibited a significant decrease in snatch duration and a significant increase in the number of retreats on the sixth day, and the number of snatches and the duration of retreats did not change significantly, consistent with our results in previous section (Supplementary Fig. S5a, b). We identified the same neurons across days by the principal components analysis, and checked the similarity of waveform and interspike interval histograms (ISIH) (Supplementary Fig. S5c, d; Materials and Methods). At the single-neuron level, we observed a realignment in the distribution of functional clusters within vHPC ensembles of mice after experiencing social loss (Supplementary Fig. S5e). Specifically, the number of neurons in Cluster 1 decreased, with 67.7% maintaining the same response as before defeat, 16.1% transitioning to the response pattern of Cluster 2, 6.4% to Cluster 3, and 9.6% becoming non-responsive to the event. Neurons in other clusters also exhibited similar realignment, with ～80% of neurons maintaining their original response patterns to events, while a small subset transitioned to the response patterns of other clusters. Of note, Cluster 5, the non-responsive cluster, showed no transformation in reactivity after social defeat (Supplementary Fig. S5f). These data suggest that HL treatment alters the reactivity and response patterns of functional clusters of vHPC neurons to competitive events.

### Ventral hippocampus dynamics encode behavior strategy during competition process

**Fig. 3:**
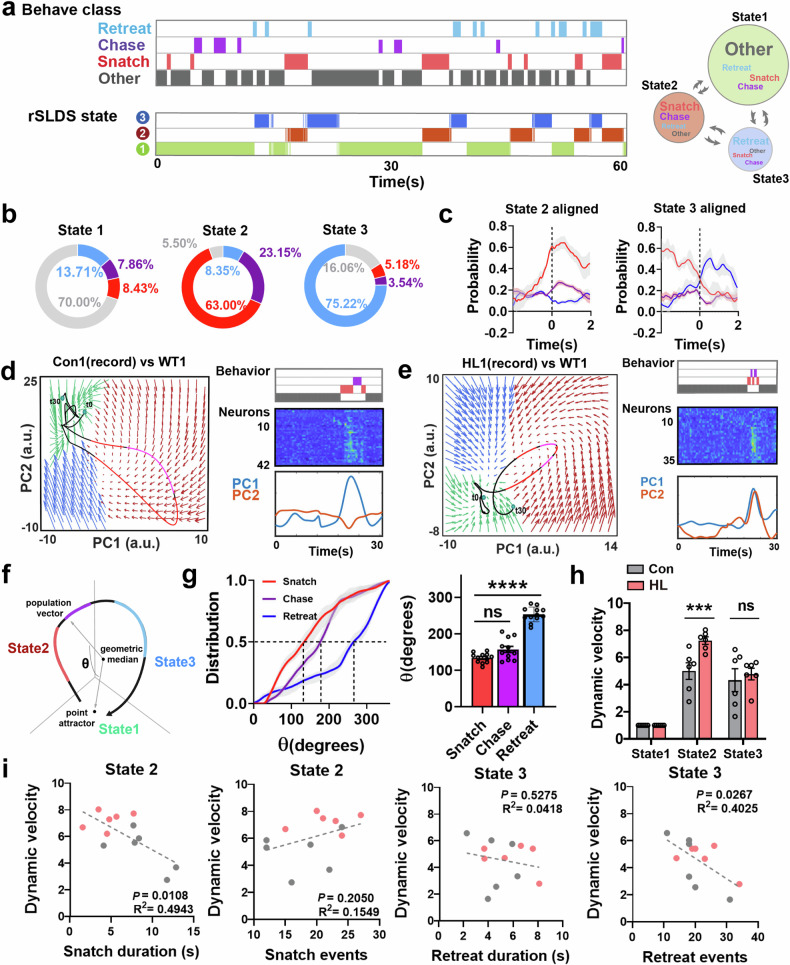
Historical loss remodels the rotation dynamics of vHPC during food competition. **a** Left, a comparison of rSLDS states with annotations of food competition behaviors. Right, state transition diagram derived from the empirical transition probabilities. **b** Composition of behaviors within rSLDS states. **c** Probability of specific behavioral class aligned to the onset of state 2 and 3 (*n* = 6 mice for each group). **d**, **e** Left, the rotational population trajectories in neural state space of mouse vHPC neurons during competition episodes, and the trajectories are color-coded to correspond with distinct behavioral categories observed in the recorded mice. Green flow field, state 1; Red flow field, state 2; Blue flow field, state 3. Right, the corresponding sequential activity of vHPC neurons during competition episodes. **f** Calculation of the rotation angle (θ) aligned to the point attractor in state 1 during competition episodes. **g** Left, empirical cumulative distribution of rotation angle (θ) for various behavioral class (*n* = 12 mice). Right, quantification of 50% distribution point of rotation angle (θ) for various behavioral classes (****P < 0.0001, *n* = 12 mice). **h** Dynamic velocity of Con and HL mice in different discrete states, using the dynamic velocity of state 1 as a baseline (***P < 0.001, *n* = 6 mice for each group). **i** Relationship between the dynamic velocity of discrete state and the performance of specific behavioral class. State 2/Snatch duration, *P* = 0.0108, *R*^2^ = 0.4943; State 2/Snatch events, *P* = 0.2050, *R*^2^ = 0.1549; State 3/Retreat duration, *P* = 0.5275, *R*^2^ = 0.0418; State 3/Retreat events, *P* = 0.0267, *R*^2^ = 0.4025; *n* = 12 mice.

Single-cell analysis revealed the reactivity of the vHPC to specific competitive behaviors, but there is a lack of insights into the population information. Therefore, we employed a previously reported unsupervised dynamic systems approach^16–18^. This approach allowed us to fit the vHPC neural activity with a recurrent switching linear dynamical system (rSLDS) model (Fig. 3a). The system contains two components: the discrete states, which segment the state space; and the continuous latent variables, which describe population activity. We determined the states and dimensionality of the model by cross-validating the evidence lower bound (ELBO) and Bayesian information criterion (BIC) with different hyperparameters (the number of states and dimensions) for each mouse (Supplementary Fig. S6a, b), Con mice and HL mice did not show any differences at the hyperparameter level (Supplementary Fig. S6h, i). We found that the best-fit models captured at least 90% of the variance in neural activity (Supplementary Fig. S6c). The accuracy of iteration in this model was assessed by the evidence lower bound of the data (Supplementary Fig. S6d). The population activity in the state space is partitioned into three discrete states, each with its own independent dynamics matrix to capture population activity within that state (Supplementary Fig. S6e). In the model, neuronal activity is simplified into a set of latent variables (dimensions) that make up a low-dimensional state space (Supplementary Fig. S6f). The dynamics matrix of discrete state determines the temporal evolution of population activity, while its parameters provide insights into the dynamic properties of that particular state.

Next, we performed retrospective alignment of the fitted discrete state with the behavioral annotations and found that the probability of snatch and chase significantly elevated during discrete state 2, while the probability of retreat significantly elevated in state 3 (Fig. 3b, c). We conducted model fitting on both Con mice and HL mice, which indicated that the relationship between states and behaviors was consistent across both groups (Supplementary Fig. S6j–o).

These data reveal the relationship between behavior and the discrete states. To further explore the deeper connections within this relationship, we employed state-specific generalized linear model (State-GLM) for each mouse^19,20^. Utilizing the previously inferred discrete states through rSLDS, along with recorded mouse behaviors, we trained multiple sets of multinomial GLMs to predict four behavioral patterns in food competition from the time series of covariate cues. The covariate cues are derived from two sources: one part comes from the continuous latent variables (Dim1–Dim6) inferred by rSLDS, and the other part consists of critical behavioral cues (distance and facing angle) in food competition. The modeling framework of this model is similar to that reported in previous study^19,21^, where each discrete state has its own unique multinomial GLM. For each discrete state’s GLM, covariate cues are used as inputs and processed through a linear filtering stage. There is a separate set of linear filters for each possible behavior. These filters are passed through a nonlinear step, which provides the overall likelihood of behavior occurrence by evaluating the relative probability of observing each behavioral output (Supplementary Fig. S7a). Models using data from either the Con group or the HL group of mice demonstrated high log-likelihood compared to chance model, and the decoder performance for behaviors on the test dataset was also above chance level (Supplementary Fig. S7b).

In each discrete state, while certain behaviors dominate, other non-dominant behaviors still occur within the same state. This suggests that the differences between each discrete state cannot simply be regarded as mere behavioral differences, but rather due to the GLM filters that predict the output of each state. To test this hypothesis, we generated behavior raster based on either the full State-GLM model or used output filters from only one of the three states (Supplementary Fig. S7c). This confirms several points. First, the output filters of each discrete state can predict all types of behaviors based on the input. Second, behavior prediction from any single discrete state is insufficient to capture the entire range of behavioral changes during the food competition process. How do these covariate cues influence behavior occurrence in different states? We examined the GLM weights for different behaviors in each state (Supplementary Fig. S7d). The most predictive feedback cues were strongly reweighted by state. For instance, Dim4 and Dim6 were the strongest predictors of snatch in state 2, but have much smaller weights in state 1 and 3. Conversely, Dim3 and Dim4, which are the strongest predictors for retreat in state 3, have minimal influence on prediction in state 2. Interestingly, different discrete states exhibit distinct response patterns to the same behavioral cues. In state 1 and state 2, the weight of distance for snatch is negative, indicating that closer distances more strongly predict the occurrence of snatch behavior. However, in state 3, this weight is positive, meaning that closer distances instead decrease the occurrence of snatch behavior. The retreat behavior shows similar performance; in state 1, both distance and facing angle are strong predictive cues for retreat, whereas in state 2, both distance and facing angle show low weights. In state 3, the occurrence of retreat is not strongly linked to distance, but a larger facing angle (i.e., face-to-face) more readily promotes the occurrence of retreat.

We further observed individuals’ different behavioral patterns when they are in different states, even under similar circumstances (Supplementary Fig. S7e). When individuals are in state 1, they exhibit a high occurrence rate of ‘Other’ behaviors across most facing angles and distances. In state 2, close proximity from opponents triggers the individual’s snatch behavior, while location behind the competitor leads to a high occurrence rate of chase behavior. When in state 3, close proximity from opponents results in a high occurrence rate of retreat behavior instead of snatch. This indicates that individuals adopt different behavioral strategy patterns in response to similar situations depending on the discrete state they are in.

### Historical loss weakens competitive behavior by influencing transitions between discrete states

To visualize the temporal evolution of discrete state during competition, we used principal component analysis (PCA) to plot its 2D flow field (Fig. 3d, e; Supplementary Fig. S8a). PCA analysis revealed that different principal components are constituted of unequally weighted latent variables in rSLDS model (Supplementary Fig. S8b) and the first two principal components (PC1 and PC2) accounted for 75% of the total variance in vHPC neuronal activity (Supplementary Fig. S8c). The 2D flow field of the PCA space showed that vHPC activity exhibited rotational neural dynamics, and the activity in food competition task displayed similar trajectories in different trials (Fig. 3d, e; Supplementary Fig. S8d–f).

By computing the angle (θ) between the position of the point attractor of state 1 and the population neural activity vector (Materials and Methods), we assessed the association between the phase of the rotation trajectory and the behavior (Fig. 3f; Supplementary Fig. S9b, d). We found that, during a rotation, different behaviors tend to occur at different rotational angles of the population activity vector. For example, snatch behavior starts to appear around 30 degrees and reaches its 50% distribution point around 150 degrees, and the chase behavior exhibits similar characteristics. On the other hand, the retreat behavior starts to rapidly increase in the latter half (Fig. 3g). Both Con mice and HL mice share similar neural representation (Supplementary Fig. S9a, c), and their angle and behavior occurrence distributions show no difference (Supplementary Fig. S9e, f). Rotational dynamics rely on the sequential activity of neurons, so we calculated its sequentiality index and found that the sequentiality index of the data was significantly exceeded that of shuffled data or random matrices of comparable sizes (Supplementary Fig. S9g). These rotational dynamics exhibit that the neural activity of vHPC switches between three discrete states and evolves continuously during food competition, corresponding to constantly varying behaviors.

How does such a neural dynamic system reflect the competitive level? To study this, we categorized the opponents into four levels of competitive ability, from low to high (C4 to C1), based on their performance in food competition. Different discrete states represent distinct behavioral strategies with state 2 indicating an individual’s active engagement in competition, while state 3 represents an avoidant strategy. The transitions and maintenance of these discrete states clearly influence an individual’s performance in competition. We then examined the differences in the transitions of discrete states when the recorded mice faced each opponent. We retrospectively calculated the average transition matrix of discrete states over the entire competition. Firstly, we found that individuals exhibited different discrete state transition probabilities when facing opponents with varying competitive abilities, particularly in the self-transition probability of S2–S2 (state 2–state 2) and the transition probability from S2–S3 (state 2–state 3) or S2–S1 (state 2–state 1), both in Con or HL mice. When facing stronger opponents, individuals exhibit a higher S2–S2 self-transition probability, as well as a higher S2–S3 transition probability and lower S2–S1 transition probability (Supplementary Fig. S10a, b). We also found that, when facing the same opponents, HL mice exhibited a lower self-transition probability in S2–S2 and a higher transition probability from S2–S3 compared to Con mice (Supplementary Fig. S10b). State2 is associated with snatch behavior. Whether these different state 2 transition probabilities affect an individual’s snatch behavior during competition remains unclear. To answer this hypothesis, we analyzed the snatch duration of individuals when they face opponents with varying levels of competitiveness. We found that when facing stronger opponents, the duration of snatch standoffs was longer with higher S2–S2 self-transition probability (Supplementary Fig. S10c) both in Con and HL groups. Additionally, compared to Con mice, HL mice exhibited lower levels in snatch duration and S2–S2 self-transition probability. We also noticed a positively correlated binomial relationship between the snatch duration and S2–S2 self-transition probability (Supplementary Fig. S10d).

We also noted that as the snatch duration increases, the rate of change in the state 2 self-transition probability differs between Con mice and HL mice, although the S2–S2 self-transition probability also increases with the duration of the snatch, but HL mice reached their plateau phase more quickly (Supplementary Fig. S10d). With the level of competition increases, HL mice reach their upper limit earlier compared to Con mice (Supplementary Fig. S10c). We can explain this difference from the perspective of dynamic systems. Dynamic velocities reflect the rate of variation in the latent continuous variables; the low dynamic velocities indicate that variable changes occur more slowly and the system tends to maintain its current state. We classified the dynamic velocities of states for different individuals into four levels, from low to high (DV1 to DV4). We found that the higher dynamic velocities showed a lower probability of self-state persistence (Supplementary Fig. S10e) and that mice with high dynamic velocities exhibited shorter state dwell timesteps (Supplementary Fig. S10f). This indicates that mice with lower dynamic velocities in state 2 have longer maximum state 2 dwell-time, and longer state 2 dwell-time is equivalent to higher S2–S2 self-transition probabilities, corresponding to longer snatch durations from an external behavioral perspective.

To test this hypothesis, we calculated the dynamic velocities of discrete states in HL mice and Con mice. We found that, compared to Con mice, HL mice demonstrated higher dynamic velocity in state 2 with no significant difference in dynamic velocity in state 3 (Fig. 3h). We then tested whether these differences are responsible for the contrasting competitive abilities between Con and HL mice. By calculating the dynamic velocities of each state with state-specific dynamic matrices, we found that the dynamic velocity in state 2 exhibited a negative correlation with the duration of snatch behavior, indicating that the dynamic velocity in state 2 determines the maintenance of active behavior. Interestingly, the dynamic velocity of state 3 did not influence the duration of retreat, but rather affected the frequency of retreat occurrences (Fig. 3i). This observation could be attributed to that, for retreat, the occurrence is more important than its duration.

We have uncovered the dynamic processes in mouse ventral hippocampus during competitive interactions. Through studying the relationship between transitions among discrete states and competitive performance, we found that historical loss leads to lower competitiveness by disturbing the transition of discrete states associated with proactive behavioral strategies.

### Molecular mechanisms underlying the effects of historical loss

The intricate interconnections between neurons form the structural foundation for neural representations^22^, and neurotransmitter receptors play a unique role in mediating these interactions^23^. Therefore, we performed a transcriptomic analysis of the hippocampus in mice with or without HL. We specifically focused on genes associated with the glutamatergic system, as they are known to play crucial roles in modulating neuronal excitability within the hippocampus^24–28^. We found that the expressions of numerous glutamate receptor-related genes, such as *glutamate ionotropic receptor AMPA type subunit 1* (*Gria1*), *glutamate ionotropic receptor NMDA type subunit 2a* (*Grin2a*), *glutamate receptor-associated protein 1* (*Grina*) and *glutamate metabotropic receptor 4* (*Grm4*), were changed in HL mice (Supplementary Fig. S11a). Notably, the downregulation of *Grina* is particularly significant (Supplementary Fig. S11b, c). These results suggest that hippocampal *Grina* downregulation may contribute to the competitive submissive behavior in mice. With immunofluorescence and western blotting analyses, we further investigated the regional specificity of the differences in Grina expression between HL and Con mice. Our findings indicate that the reduction in Grina expression in HL mice was notably pronounced in the vHPC rather than the mPFC (Supplementary Fig. S11d–g).

Grina, also known as Tmbim3, is an evolutionarily conserved transmembrane BAX inhibitor motif (Tmbim) protein that has been shown to protect cells from apoptosis^29^. However, the expression pattern and physiological functions of Grina in the brain are poorly understood. We found that the Grina protein was widely distributed in the whole brain, including the thalamus (Tha), hindbrain (Hb), striatum (Str), entorhinal cortex (Ec), mPFC, cerebellum (Ceb), and hippocampus, but a peak level was detected in the hippocampus (Supplementary Fig. S11h), consistent with a previous report^30^. Double immunofluorescence staining results suggest that Grina was mainly expressed in NeuN-positive neurons but not GFAP-positive astrocytes or IBA-1-positive microglia (Supplementary Fig. S11i, j). These data suggest a potential involvement of *Grina* alterations at the vHPC neurons in controlling social competition.

### Loss of *Grina* disrupts dynamics in competition

To examine the role of *Grina* in the reduced social competition in the HL mice, we generated conditional *Grina*-KO mice with CRISPR/Cas9-mediated gene editing strategy^31^. By targeting exons 2–7 of the *Grina* gene on mouse chromosome 15, loxP sites were inserted flanking these exons (Supplementary Fig. S12a), and this generated a targeted allele that can be conditionally knocked out upon Cre recombination. Then we specifically knocked out *Grina* in the ventral hippocampus neurons by injecting Cre virus (AAV2/9-hsyn-Cre-RFP) or control virus (AAV2/9-hsyn-RFP) into the ventral hippocampus of *Grina* flox mice (Supplementary Fig. S12b). Immunofluorescence demonstrated a significant decrease in the expression level of *Grina* in the virus-infected neurons (Supplementary Fig. S12c, d). Also, insertion of loxP sites showed no impact on mouse development, as evidenced by comparable body sizes to wild-type mice (Supplementary Fig. S12e).

We then assessed the behavioral changes in *Grina* cKO mice and found that these mice displayed a lower social competition, as shown by reduced winner times in the tube test when they were either single-housed (Supplementary Fig. S13a) or group-housed with wild-type mice (Supplementary Fig. S13b, c). Moreover, in the warm spot test with four cage mate mice (3 WT mice and 1 cKO mouse) competing for a warm corner in a cage with an ice-cold floor (Supplementary Fig. S13d), the cKO mouse occupied the shortest amount of time in the warm corner in a 20-min test, indicating lower social competitiveness of this mutant mouse (Supplementary Fig. S13e, f). The amount of time that each mouse occupied the warm spot was highly correlated with its tube test winner times (Supplementary Fig. S13g), confirming the reduced social competition of the cKO mice. Taken together, these results indicate that *Grina* cKO mice exhibited competitive submissive behaviors even without historical loss. We also found that the performances of cKO mice were similar to those of Con littermates in multiple behavioral assays, with a lack of anxiety-like symptoms (elevated plus maze), and no depressive-like symptoms (sucrose preference test) (Supplementary Fig. S13h, i), indicating that absence of *Grina* did not induce neuropsychiatric disorders. Similarly, we monitored the 24-h food intake behavior of cKO mice and found that, compared to control mice, the appetite of cKO mice was not affected (Supplementary Fig. S13j, k).

**Fig. 4:**
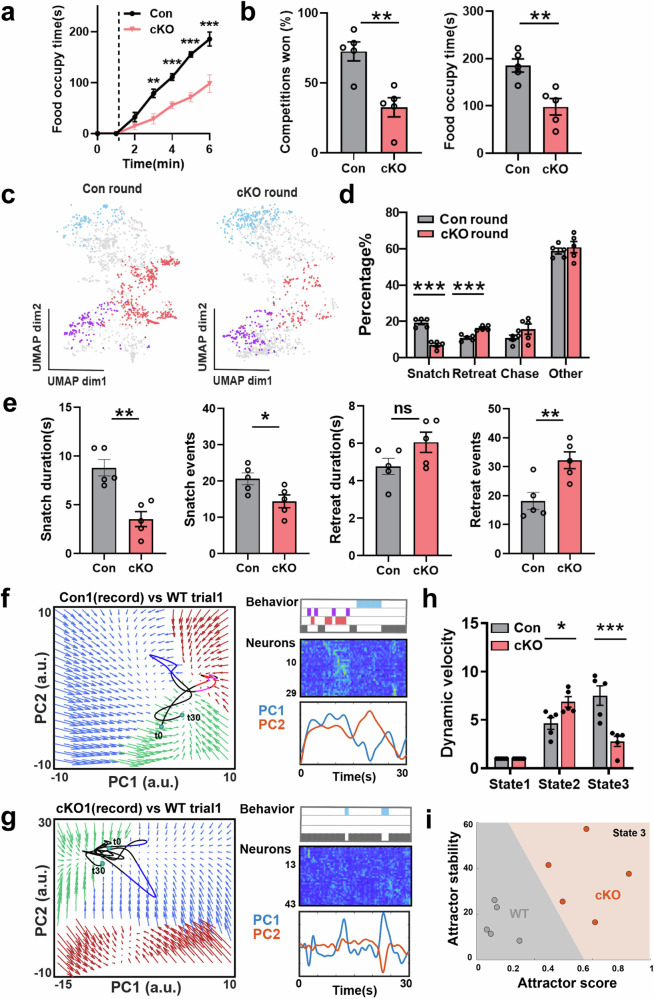
Genetic ablation of *Grina* in vHPC leads to reduced social competitiveness through remodeling of neural dynamics. **a** The cumulative time of food occupation during the 6-min food competition task in Con and cKO mice (**P < 0.01 and ***P < 0.001, *n* = 5 for each group). **b** Left, in the food competition task, the winning rate of mice in each bout of scramble during competition task (*P* < 0.01, *n* = 5 for each group). Right, total food occupancy time of mice in the food competition task (*P* < 0.01, *n* = 5 for each group). **c** The UMAP plot of video clips color-coded by behavioral classification in Con round (Con mice as challengers) and cKO round (cKO mice as challengers) during a representative food competition trial. **d** Percentage of frames that correspond to a specific behavioral class out of the total frames in Con round and cKO round, *n* = 5 for each group. **e** The duration and frequency of specific behavioral class in Con and cKO round. Snatch duration, *P* = 0.0017; Snatch events, *P* = 0.0331; Retreat duration, *P* = 0.1013; Retreat events, *P* = 0.0097; *n* = 5 mice. **f** Left, the rotational populatio*n* trajectories in neural state space of Con mouse 1 vHPC neurons during competition episodes. The trajectories are color-coded to correspond with distinct behavioral categories observed in the recorded mice. Green flow field: state 1; Red flow field: state 2; Blue flow field: state 3. Right, the corresponding sequential activity of vHPC neurons during competition episodes. **g** Same as **f** but cKO mice 1. **h** Dynamic velocity of Con and cKO mice in different discrete states. The dynamic velocity of state 1 was used as a baseline (***P < 0.001, *P < 0.05, *n* = 5 mice). **i** Scatter plot for attractor stability score vs line attractor score of state 3 separates Con and cKO mice.

In the food competition task, cKO mice showed reduced cumulative occupancy time (Fig. 4a) and lower win rate (Fig. 4b). Compared to the control mice, cKO mice showed fewer snatch behaviors, more retreat behaviors, and comparable chase or other behaviors when acting as challengers (Fig. 4c, d). Similar to mice with historical loss, cKO mice exhibited shorter snatch durations and more retreat events (Fig. 4e). These findings suggest that mutant mice displayed submissive behaviors in competitive situations, even without prior social loss.

We also performed in vivo electrophysiological recordings during the food competition task. Compared to control mice, the cKO mice exhibited a decreased proportion of cluster 1 neurons (responding to snatch) and cluster 4 neurons (showing a decline in response during retreat), while the proportion of cluster 2 neurons (displaying an increased response to retreat) increased (Supplementary Fig. S14a). Some neural clusters also exhibited significant changes in response amplitude (Supplementary Fig. S14b–e). The firing rate variance distribution of all functional groups of neurons in the cKO mice exhibited a leftward shift, particularly in cluster 1 (Supplementary Fig. S14f). In addition, the (local field potentials (LFPs) analysis revealed that cKO mice displayed an increased alpha rhythm and a decreased low gamma rhythm during free-moving compared to Con mice (Supplementary Fig. S15a, b), and the difference displayed velocity independence (Supplementary Fig. S15c). Additionally, we observed a significant reduction in miniature excitatory postsynaptic current (mEPSC) amplitudes and AMPA/NMDA ratios in hippocampal neurons of cKO mice (Supplementary Fig. S15d–h). These findings suggest that the KO of *Grina* gene has a notable effect on the pattern of neuronal synchrony and neuronal synaptic function in the vHPC.

Interestingly, in cKO mice, state 3, manifested as a linear attractor, showed significant extension of the period of population activity (Fig. 4g). Additionally, cKO mice exhibited slightly higher dynamic velocity in state 2 and very low dynamic velocity in state 3 (Fig. 4h). These findings suggest that during food competition, cKO mice primarily adopted a passive behavioral strategy represented by state 3, which aligns with the increased number of retreat trials observed in behavior analysis. To investigate the changes of state 3 between cKO mice and Con mice, we assessed the line attractor scores and stability of state 3. Interestingly, cKO mice exhibited higher line attractor scores and greater attractor stability in state 3 (Fig. 4i), indicating a greater likelihood of remaining in state 3 and maintaining stability compared to Con mice. This increased stability of state 3 corresponds to the observed higher frequency of retreats in cKO mice.

These findings suggest that the alterations in AMPA/NMDA receptor-mediated synaptic transmission may be associated with the changes in neural dynamics observed in *Grina* cKO mice and contribute to their lower social competitiveness.

### Real-time restoration of vHPC *Grina* expression enhances the social competition

**Fig. 5:**
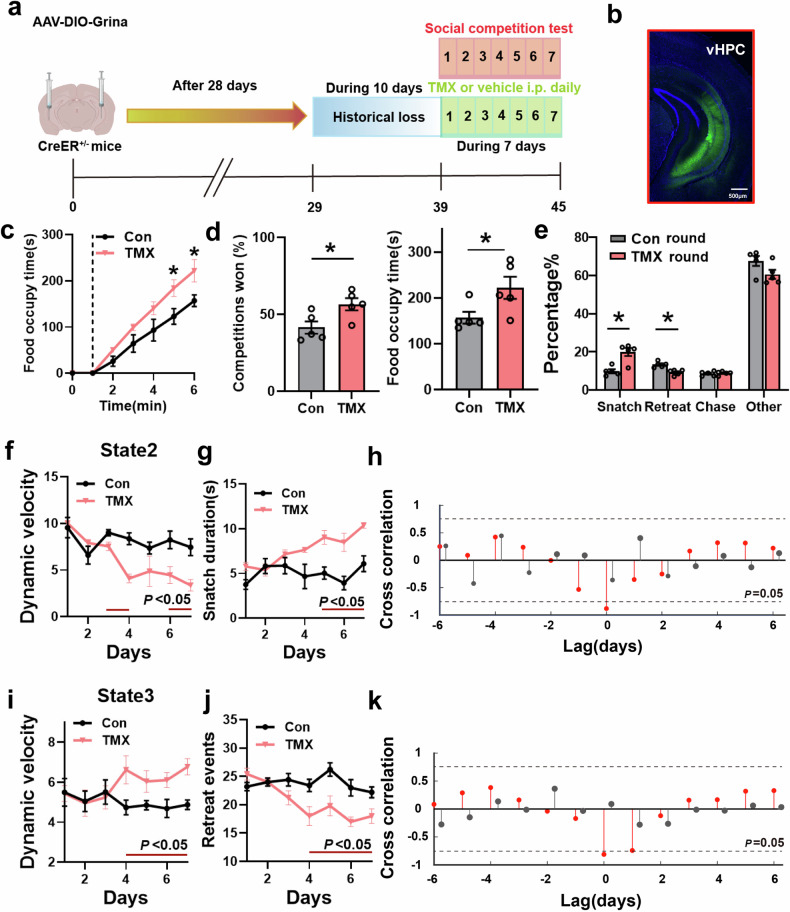
Restoration of vHPC *Grina* effectively elevates the social competitiveness in HL mice. **a** A diagram of the experimental flowchart. Neuronal and behavioral evaluations were performed after the administration of TMX. TMX was administered via intraperitoneal injection (i.p.). **b** A representative fluorescence image showing the expression of *Grina* in vHPC neurons following TMX-injection. **c** The cumulative time of food occupancy during the food competition trials of the vehicle-i.p. (Con) and TMX-i.p. mice at day 7 (*P < 0.05, *n* = 5 for each group). **d** Left, the winning rate of mice in each bout of scramble during competition task at day 7 (*P* < 0.05, *n* = 5 for each group). Right, total food occupancy time of mice in the food competition task at day 7 (*P* < 0.05, *n* = 5 for each group). **e** Percentage of frames that correspond to specific behavioral class out of the total frames that in Con round (Con mice as challengers) and TMX round (TMX mice as challengers) at day 7, *n* = 5 for each group. **f**, **g** Continuous changes in dynamic velocity in state 2 (**f**) and snatch duration (**g**) after TMX or Con (vehicle) administration (*n* = 5 for each group). **h** The cross-correlation between changes in competitive behavior (snatch duration) and changes in neural representations (state 2 dynamic velocities). Red, TMX; Gray, vehicle. **i**–**k** Same as **f**–**h**, but for dynamic velocity in state 3 and retreat events.

The aforementioned observations suggest a correlation between *Grina* expression and neural dynamics in the vHPC. However, they do not provide information on the extent to which changes in neural dynamics causally influence the mouse’s social competitive behavior. To address this question, we employed CreER-mediated control of *Grina* expression and simultaneously tracked animal’s neural activity and competitive behavior. We injected AAV2/9-SYN-DIO-Grina-EGFP (10^12^ IU/mL, 200 nL) bilaterally into the vHPC of CamKII-CreER mice and subjected the mice to loss. Then, tamoxifen (TMX) or vehicle (Control) was administered to restore *Grina* expression in the vHPC, and neural activity and competitive behavior were monitored (Fig. 5a). Mice that received TMX injection exhibited gradual restoration of *Grina* in the vHPC (Fig. 5b; Supplementary Fig. S16a, b), as well as enhanced social competition at day 7 (Fig. 5c–e; Supplementary Fig. S16c–e). After the day 7, we co-housed a mouse from TMX group with three mice from Con (vehicle) group for two weeks and found that the restoration of *Grina* also affected the social hierarchy distribution after co-housing for 2-week (Supplementary Fig. 16f, g). This finding suggests that specific activation of *Grina* expression contributes to the enhancement of social competitiveness and influences the establishment of social hierarchy to some extent.

We next evaluated the dependency between changes in competitive behavior and neural dynamics (by examining their daily progression). On the third day, the dynamic velocity of state 2 decreased significantly compared to the Control group (Fig. 5f), while on the fourth day, the dynamic velocity of state 3 significantly increased (Fig. 5i). Furthermore, the duration of snatch significantly increased on the fifth day of continuous TMX injection compared to the Control group (Fig. 5g), and changes in the counts of retreats were observed starting from the fourth day (Fig. 5j). We also found a significant negative correlation between the neural dynamics (the dynamic velocity of corresponding states) and the performance in food competition (duration or frequency of snatch/retreat) over time by cross-correlating the quantified parameters as a dot product of time changes. Notably, changes in neural dynamics and competitive behavior exhibited no delay in peak times, indicating a direct influence of neural dynamics on the competitive performance of mice in food competition. In contrast, the Control mice treated with the solvent did not display any variations in neural dynamics or competitive behavior over time, and there was no temporal relationship observed between the two time sequences (Fig. 5h, k). These findings suggest that the enhancement of competitive ability resulting from TMX treatment relies on the expression of *Grina* in the vHPC.

At the single-neuron level, mice with restored *Grina* exhibited a higher proportion of cluster 2 neurons, and a lower proportion of cluster 4 neurons compared to the Control mice (Supplementary Fig. S17a), the proportional change suggests that *Grina* plays a complex role in the neural network structure of the vHPC. Furthermore, in mice with restored *Grina*, cluster 1 and cluster 3 neurons displayed greater responsiveness to snatch behavior (Supplementary Fig. S17b, d), cluster 2 exhibited reduced responsiveness to retreat behavior (Supplementary Fig. S17c), and cluster 4 showed increased responsiveness to snatch events (Supplementary Fig. S17e), suggesting that restoration of *Grina* in the vHPC reversed the loss of response specificity to snatch/retreat behavior for functional groups of neurons. Additionally, a rightward shift in the variance of firing rates was observed in cluster 2 neurons between the Control and TMX groups (Supplementary Fig. S17f), suggesting that the recovery of *Grina* also altered the intrinsic excitability of neurons.

## Discussion

Competitive behaviors within animal groups exhibit a high degree of complexity, as individuals need to integrate external social, economic, and environmental factors^32–34^, and also to consider their own social attributes and physiological states^35^, in order to exhibit different social behaviors in response to the current circumstances. During the competitive process, these fast-changing factors require individuals to flexibly adopt appropriate behavioral strategies to adapt to different situations, yet the neural mechanisms underlying these processes remain unclear. To address this question, previous studies have developed different behavioral paradigms for the competition of liquid reward or food^11,12^. Here, our study focused on the natural basis of food competition which is a ubiquitous scenario for resource competition. Through tracking and semantic segmentation of food competition behaviors, we observed that a mouse’s ability to occupy food resources is a comprehensive outcome resulting from a sequence of positive and negative behaviors exhibited by individuals during food competition. Moreover, historical loss pulls down the endurance of positive behaviors in mice.

The hippocampus is one of the key brain regions that play important roles in regulating emotional and cognitive behavior^36^. It regulates the individuals’ reward-seeking behaviors by modulating the reward learning and motivation^37,38^. It also participates in avoidance behavior^39^, balancing behavioral responses to conflicting stimuli in a state- and context-dependent manner to determine approach or avoidance strategies^40,41^. Furthermore, the vHPC is thought to play a significant role in behavioral flexibility^42^, linking external stimuli with internal drive state to promote the selection of adaptive behavioral responses^43^. These findings fit our conclusions that food-driven social competition is a complex behavioral process, where individuals must balance the valence of rewards and the threat of conflicts to make adaptive approaches or avoidance decisions. By mapping and visualizing the population activity of vHPC neurons, we provide evidence for the function of the vHPC in complex social interactions. Here, by performing neuronal recordings and multiple-instance tracking on mice during the food competition task, we find that population neural activities in the vHPC capture the temporal structure of mouse’s mental process during competition. The rSLDS analysis of vHPC neural activity during the food competition process revealed a recurring rotational dynamics, exhibiting cyclic activity patterns throughout the continuous competition, and that the behavioral strategy during competition can be decoded based on the population activity of vHPC.

Rotational dynamics have been found in many brain regions, each encoding different functions. For example, in the motor cortex^44^, they encode motor control; in the prefrontal cortex, they encode decision-making^45^; and in the subicular complex, they are involved in spatial information processing^46^. Although the content they encode varies, their latent dynamics all form circles or closed loops^47^. This is why they are referred to as rotational dynamics. The reasons behind the formation of these circles or loops are complex, but in some aspects, this rotation represents the cyclical and continuous sequential activity of neuronal populations^48^.

Another part of rotational dynamics is the dynamical system. At the heart of this theory are differential equations that express the temporal dynamics of a system’s state variables according to the physical laws governing the system^49^. The system’s differential equations prescribe a flow — the temporal change of the system from each of its possible states. Such flows link to form orbits, yielding time series for each of the states. These internal dynamics are visualized as arrows in the neural flow field, depicting the movement vectors of points within the state space. Interestingly, external input can perturb the trajectory, but the trajectory’s evolution is still determined by the system’s internal dynamics. This is also the core argument of Kennedy's report^16^, a linear attractor in the hypothalamus. In the neural flow field of the VMHvl, the points where vector length is at a minimum (slow points) form a trough-like structure in the dynamics landscape, along which neural activity progressed slowly as aggression escalated. Notably, this progression along the trough-like structure does not require external input for support. On the contrary, external stimuli pushed VMHvl activity away from the trough of the line attractor, but the signal decayed relatively quickly, the system re-entered the line attractor at nearly the same point. This is why dynamical system is considered to be a relatively independent and self-consistent system; while it does receive external input, its characteristics largely depend on its own intrinsic dynamics. This is what we observed in the vHPC: external input can influence the bifurcation of neural trajectories (toward state 2 or state 3), but the duration spent in state is still determined by the dynamical matrix.

Likewise, Kennedy's report also mentions the rotational dynamics in the mPOA, although it does not hold a prominent position. The phase of the rotations was correlated with progression through sniffing, mounting, and intromission. Compared to linear attractors, rotational dynamics appear simpler, representing a cyclical sequence of activities. During vHPC, this process corresponds to the transition of behavioral strategies during the course of competition, from peaceful coexistence to active engagement in confrontation, and then to passive retreat. Interestingly, we found a strong correlation between mice’s competition performance and the dynamic velocity of their positive and negative states, which explains the individual differences in competition performance and the neural correlations within the vHPC.

The neural encoding of social behavior that we observed in the vHPC may reflect its unique neurochemical and cellular structural features. The vHPC exhibits high transcriptional heterogeneity, with a diverse population of glutamatergic and GABAergic neurons^50,51^. The glutamatergic neurons, by their excitatory recurrent connections, enable the persistence of network activity, and changes in glutamatergic synaptic efficacy directly influence the maintenance of this persistent activity^52^. The GABAergic neurons, in particular, may provide the basis for mutual inhibition between action-specific subgroups, generating effects similar to winner-takes-all dynamics or feedforward inhibition^22^, to control the switching between consecutive behavioral strategies. Further research is needed to delve into the transcriptional characteristics and cell types of these heterogeneous neurons. Investigating these aspects will provide a more comprehensive understanding of the underlying mechanisms associated with the observed rotation dynamics in the vHPC.

Social competition is a complex decision-making behavior involving multiple brain regions^35,53^. In the current competition models, mPFC plays an undisputedly central role, and it identifies social states and determines the outcome of social competition^10–12^. Many brain regions interconnected with the mPFC have also been reported to play roles in social competition: mediodorsal thalamus (MDT) was considered to regulate winner-effect^4^; hypothalamic ventral premammillary nucleus (PMv) organizing goal-oriented aggression is used to establish social rank^54^; lateral habenula (LHb) participates in social status loss and depressive behaviors^14^. While the ventral hippocampus is currently associated with social interactions, its role in social competition remains unclear. As we observed, the vHPC exhibits progressive rotational dynamics to switch behavioral strategies during competition. However, the dynamic system of this single brain region cannot fully explain social competition. For example, the point attractor is formed in discrete state 1. From a dynamical perspective, when neural activity is in state 1, it gravitates toward the point attractor and remains stable there in the absence of external forces, corresponding to the situation where no competition is taking place. However, we found that at the onset of competition, neural activity transitions from state 1 to state 2. This trajectory would not occur if we only consider the dynamics of the vHPC, indicating that the vHPC receives motivation input from other brain regions, pushing neural activity away from state 1. Meanwhile, the binary controller determining the regression direction of the population activity vector in state 2 signifies the decision-making process regarding the competition’s outcome. These functions require the support of upstream/downstream brain regions in conjunction with the vHPC to collectively execute a social competitive behavior. As previously reported, the prefrontal cortex determines the direct outcome of social competition^10–12^, which is a downstream region receiving rich projections form the vHPC^55,56^. Also, the basolateral amygdala (BLA), a brain region with bidirectional projections to the ventral hippocampus^57,58^, is involved in reward processing^59,60^ and also is a candidate for inputting motivational factors in competition^61,62^. Thus, we hypothesize that vHPC is located in an intermediate position for integrating information and providing strategy biases, and plays a critical role in providing valence inputs for behavioral strategies.

Given the current lack of clarity regarding the transcriptional features and cell types of these heterogeneous neurons, the use of non-specific genetic methods (such as Optogenetics or DREADDs) for extensive interventions in the vHPC is highly inefficient and inconclusive, as it may disrupt the sequential activation of neural clusters. To address this concern, we performed the transcriptomic analysis in the vHPC and identified a novel glutamate receptor-associated protein, Grina, in the regulation of submissive behaviors in HL mice.

As a highly expressed hippocampal ion channel-binding protein, *Grina* has been implicated in several hippocampus-associated psychiatric disorders, including schizophrenia and epilepsy^63,64^. The Grina protein is located in the Golgi apparatus^26^, ER^65^ and plasma membrane^66^. It plays an important role in endosome-to-Golgi retrieval^67^ and the maintenance of ER calcium homeostasis by interacting with inositol trisphosphate receptors to protect cells against ER stress-related apoptosis^65^. Because disruption of ER is involved in many behavioral and cognitive disorders in mice^68,69^, we propose that *Grina* plays a crucial role in behavioral changes in HL mice. We find that the expression of *Grina* was dramatically reduced in the ventral hippocampus of HL mice, and deletion of *Grina* by genetic approach led to lower social competitive behaviors by remodeling the neural dynamics in ventral hippocampus. Furthermore, restoration of *Grina* in the ventral hippocampus restored the social competition in mice, suggesting that targeting *Grina* might be a suitable intervention for subordinates resulting from the failure experience.

While our study reveals a novel role for *Grina* in social competition after HL in the current study, some intriguing questions remain to be elucidated. For instance, our data showed that the deletion of *Grina* resulted in the obvious inhibition of AMPA receptor-mediated mEPSCs but not NMDA receptor-mediated mEPSCs. However, Grina was first identified from the NMDA receptor complexes^70^, suggesting a direct interaction between Grina and the NMDA receptors. It is still unclear whether Grina and AMPA receptors physically interact, and how Grina affects AMPA receptor function.

In summary, our findings demonstrate that prior social loss causes a reduction in the *Grina* in the hippocampus, which changes the neural dynamics and results in a low competitive performance in mice. *Grina* restoration effectively rescues neuronal reactivity and reinforces the social competition in mice, suggesting that *Grina* might be a promising novel therapeutic target for treating distressful social experiences-caused psychiatric disorders.

## Materials and methods

### Animals

*Grina* flox mice were generated by using the CRISPR-Cas9 system (Cyagen Biosciences Inc., Suzhou, China). The *syn2b* knockdown mice were generated as described in our previous study^13^. The CamkII-CreER mice (012362, Jackson Labs) were purchased from JAX. All mice used in this study were male mice with C57BL/6 backgrounds. All animals were housed under standard housing conditions with free access to food and water and bred in the experimental animal central of Tongji Medical College, Huazhong University of Science and Technology. All animal experiments were carried out according to the “Policies on the Use of Animals and Humans in Neuroscience Research” revised and approved by the Society for Neuroscience in 1995, the ethics approval document code is 2019S1874.

### Historical Loss

Historical loss is based on the tube test, with certain modifications. Firstly, mice in the HL group or Con group were group-housed in their respective cages, four mice per cage, and both groups underwent training to traverse the tube alone before the HL training to ensure familiarity with tube crossing. Subsequently, the mice in the HL group underwent the tube test, where their opponents were inherently higher-ranking, unfamiliar *Syn2b*-KO mice^13^, resulting in the HL mice always being defeated. This defeat process lasted for 10 consecutive days, with 5 trials daily. During this period, the *Syn2b*-KO mice were rotated to ensure a new opponent for HL mice each day. The Con group continued to go through the tube alone daily. During the HL training period, the two groups of mice did not interact. After the HL training was completed, further behavioral tests were conducted on day 11.

### Social competition behaviors

The social competition tests include the following four independent tasks as reported previously^35^. The food competition and single-housed tube test were tested on the same day, in the order of food competition and then tube test, with a 4-h interval. Warm spot and group-housed tube test was tested after mice being co-housed for 2 weeks following the completion of the other behavioral tests.

### Tube test

Tube test was performed as described previously^10^. In brief, two mice with similar body weights from different groups were placed into opposite ends of the tube and guided to meet at the center of the tube. The trial was terminated when a mouse was pushed out of the tube as the “loser” and the other as the “winner” of that trial. We used two types of tube tests. The first type, the single-housed tube test, aimed to assess the competitiveness of each mouse before hierarchy was established. Supplementary Figs. S1b, S13a, S16c utilized this method. During single-housed tube test, these two groups of mice were separate-housed and did not interact before competition test. Each test mouse was challenged five times with a novel strange control mouse and the winner times were calculated. The second type, group-housed tube test, aimed to assess the established hierarchy, Supplementary Figs. S13b, c and S16f, g utilized this method. During the group-housed tube test, we co-housed one HL/cKO/TMX mouse with three Con mice in a cage for two weeks. Then, the classic round-robin tube test was conducted, the winner times of each mouse were calculated after six repeated session tests. Before each trial, the tube was cleaned with 75% ethanol.

### Warm spot test

The test was performed as described previously^10^. Before this experiment, we co-housed one HL/cKO mouse with three Con mice in a cage for two weeks. Then, four group-housed mice (one HL/cKO mouse and three Con mice cagemates) were first placed in a cold cage (28 cm × 20 cm) for 30 min to cool down and then transferred to the test cage, where a round nest heated by coil underneath at 20 °C was placed at one corner for 20 min. The nest was 5 cm in diameter and just large enough to permit the stay of only one adult mouse. The behaviors of four mice in the test cage were videotaped, and the occupation time of the warm nest was analyzed.

### Food competition test

After historical loss process, HL group and Con group mice were subjected to two-day food restriction until they reached 80% of body weight. Mice for each pair, one from an HL cage and one from a Con cage, were selected by using a round-robin strategy, and were subjected to food competition test. Before the experiment, the pair of mice had not met each other. After that the mice are placed in home-cage (10 cm × 5 cm). An industrial camera (30 Hz) is used to shoot from the field, and the mice are allowed to explore freely for 1 min, then a 5 mg circular food pellet (Kellogg, Thailand) is placed in the center of the field for competition. Shooting ends after the food pellet has been eaten, this typically lasts for 6–8 min, and the video is provided for subsequent analysis.

### Elevated plus maze test (EPM)

The EPM was performed as previously described^71^. Briefly, the behavioral apparatus comprises four elevated arms radiating from a central platform, forming a plus-shaped configuration. Two of the opposite arms are enclosed with walls (except for the ceiling, entrance, and exit points), while the other two opposite arms remain open, except for the platform itself. During the test, mice were placed in the central area of the maze and allowed to freely explore for a specified short duration. The time spent in the walled arms was then compared to the time spent in the open arms to assess levels of anxiety or fear.

### Sucrose preference test (SPT)

The SPT was performed as previously described^72^. Briefly, the mouse cages were modified to fit two bottles. One bottle was filled with pure water, and the other was filled with 1% sucrose solution. Mice were acclimatized to the two-bottle conditions for two consecutive days. After acclimation, the mice were tested for 24 h. Then, the fluid consumption was measured as indicated by the weight loss of the bottles, and the sucrose preference was calculated as the percentage of sucrose intake/total liquid consumption.

### Western blotting assay

Tissues were homogenized, and total proteins were extracted as described previously^73^. After quantification by a BCA protein assay kit (ThermoFisher, #23225), equal amounts of proteins were separated by 10% SDS-PAGE gel and transferred to nitrocellulose membranes. After blocking with 3% milk for 1 h at room temperature, the membranes were incubated with primary antibodies at 4 °C overnight, followed by incubation with goat anti-mouse IRdye 800 secondary antibodies or goat anti-rabbit IRdye 700 secondary antibodies for 1 h at room temperature and detected using an Odyssey Imaging System (LI-COR, Lincoln, NE, USA). The results were analyzed using ImageJ software (version 1.45b). All of the primary antibodies used in this study are listed in Supplementary Table S1.

### Immunofluorescence and Immunohistochemistry

Mice were euthanized and immediately perfused continuously with normal saline and 4% paraformaldehyde solution. Brain slices (20 μm) were prepared as described previously^74^. For immunofluorescence labeling, brain sections were incubated with the primary antibodies (as listed in Supplementary Table S1) overnight at 4 °C, washed, and subsequently incubated with red or green fluorescent secondary antibodies for 1 h. Then, the slices were incubated with DAPI (1:5000, Sigma) for 10 min and imaged using a laser confocal microscope (LSM780, Carl Zeiss). For immunohistochemistry staining, the slices were incubated with 0.3% hydrogen peroxide solution for 30 min. Then, the slices were incubated with 0.5% Triton X-100 for 30 min and with 3% BSA in PBS for 30 min at room temperature. Primary antibodies (as listed in Supplementary Table S1) were incubated with slices overnight at 4 °C. Subsequently, the slices were incubated with biotin-labeled His-tagged secondary antibody (dilution ratio 1:300) at room temperature for 1 h and streptomycin-labeled peroxidase working solution (diluted 1:300, diluted in PBS buffer) at room temperature for 1 h. After staining with DAB reagent for 5–15 min, the slices were observed using a microscope (SV120, Olympus).

### RNA sequencing and analysis

Hippocampal samples were rapidly isolated from the brains of HL mice and control mice, and RNA-sequencing experiments were performed by Capitalbio Technology Corporation (Beijing, China). The differentially expressed genes were determined by |log_2_FoldChange| > 0 and *P* < 0.05. GO and KEGG enrichment analyses were performed on the Metascape website (http://metascape.org/gp/), and GSEA was performed using GSEA software.

### Surgery

The surgery was performed under a stereotaxic device (RWD life science, China) using 3% isoflurane (RWD life science, China) for induction of anesthesia and 1.5% isoflurane for maintenance. Bottom heating was used to stabilize the body temperature. After the mouse surgery was awake, it was transferred to a clean cage and the experiment was performed after 7 days of recovery.

### Virus and stereotaxic injection

Adeno-associated viruses (AAVs) for *Grina* conditional KO (AAV2/9-SYN-Cre-P2A-RFP, 2 × 10^12^ IU/mL) and the control virus (AAV2/9-SYN-P2A-RFP, 2 × 10^12^ IU/mL) were purchased from Brain Case (Shenzhen, China). Virus (300 nL) was bilaterally microinfused into ventral hippocampus (anterior/posterior = −3.3 mm, medial/lateral = ±3.15 mm, dorsal/ventral = −4.2 mm) of Grina flox mice via a cannula connected to a Hamilton micro-syringe (Reno, NV). AAVs for CreERT-mediated control of *Grina* expression (AAV2/9-SYN-DIO-*Grina*-EGFP, 2 × 10^12^ IU/mL) were purchased from Brain Case (Shenzhen, China). Virus (300 nL) was bilaterally microinfused into ventral hippocampus (anterior/posterior = −3.3 mm, medial/lateral = ±3.15 mm, dorsal/ventral = −4.2 mm) of CamkII-CreER mice via a cannula connected to a Hamilton micro-syringe (Reno, NV). In this study, 28 days after virus injection, mice were used for phenotyping assays and behavioral tests.

### TMX administration

To restore *Grina* expression in awake-behaving adult mice, TMX was administered following the protocol described by Susanne et al. ^75^. Briefly, mice weighing between 26 g and 29 g were given a daily dose of 7 mg TMX. TMX was dissolved in corn oil (Sigma Life Science) to a volume of 0.35–0.40 mL, depending on the TMX dose. The administration of TMX was performed by intraperitoneal injection using a disposable syringe (Instech). TMX administration began after HL treatment and was given for 7 d. For control comparison, vehicle was given to the virus-injected CreER^+/–^ mice.

#### Electrode implant

Mice were anaesthetized with isoflurane (induction 3%, maintenance 1.5%). Room temperature was maintained at 25 °C. The eyes were covered with erythromycin. Following craniotomy, mice were placed in a stereotaxic frame (RWD), and three stainless steel screws were attached to the skull. A steel screw was used as a ground electrode. Mice were unilaterally implanted with electrode arrays (Global Biotech Inc, Shanghai, China). The vHPC was targeted by the following coordinates relative to bregma: anterior-posterior (AP), −3.3 mm; medial-lateral (ML), ±3.15 mm; and dorsal-ventral (DV), −4.2 mm. Each electrode (Global Biotech Inc, Shanghai, China) contains eight stereotrodes and was fixed to an electrode guide. The connectors were referenced and grounded via silver wires (127 μm in diameter, A-M Systems) that tightly connected to a steel screw. All implants were secured using Super-Bond cement. During surgery, long- and short-lasting analgesic agents were injected. After surgery, mice were allowed to recover in their home cage for 7 days.

### Electrophysiological recordings in vitro

Hippocampal slices were prepared as previously described^76^ and recovered in a holding chamber containing the following (in mM): 3 KCl, 7 MgCl_2_, 26 NaHCO_3_, 1.25 NaH_2_PO_4_, 10 glucose, and 212 sucrose. mEPSC currents were recorded and analyzed as described in our previous study^13^. Hippocampal neurons were held at −70 mV to record AMPAR EPSCs and at +60 mV to record NMDAR EPSCs in the presence of CNQX (10 μΜ). Current-voltage-dependent AMPA/NMDA ratios were also determined. Data were analyzed with Mini 60 and Clampfit 10.2 software.

### Electrophysiological recording in vivo

During electrophysiological recording in vivo, electrode arrays were connected with head stage (Plexon) containing AC-coupled unity gain operational amplifiers (Plexon). Each head stage was connected with a PBX preamplifier in which the signal was amplified by 4000- to 20,000-fold and isolated using a 250 Hz lowpass filter and a 250 Hz high-pass filter. Spiking activity was isolated by time-amplitude window discrimination and template matching using an Omniplex system (Plexon).

To record the activity of ventral hippocampus neurons in the food-competition task and eliminate variations introduced by different opponents, electrode-implanted mice, both Con or HL/cKO/TMX group, were each paired with four unfamiliar WT mice from the same cage to carry out multiple food-competition tasks with electrophysiological recording. As a control, we also recorded the activity of ventral hippocampus neurons in the condition that mouse solitary foraging alone in the homecage or engaging in social interactions with its opponent in the absence of food. Single-unit spike sorting was performed using off-line spike sorter (OFSS, Plexon), in which pairwise *P* (multivariate ANOVA) statistics were used to assess unit isolation quality (*P* < 0.05). At the end of the experiment, electrolytic lesions were administered before transcardial perfusion to verify electrode tip location by using standard histological techniques.

### Real-time neural activity and competitive behavior monitoring

Monitoring of individual behavior and neural activity was consistent with aforementioned methods. During the monitoring period, the mice’s diet was controlled to maintain 80% of their body weight. The monitoring was conducted every two days. During the period, the recorded mouse was engaged in multiple food-competition tasks during each monitoring session, and its opponent was an unfamiliar WT mouse and remained fixed during monitoring period. The data from the competition was recorded for subsequent analysis, the data from each monitoring session were analyzed independently. The methods are consistent with those described above.

### Neuron tracking across days in electrophysiology recordings

Identifying the same neurons cross days is challenging, because of the complication by non-rigid movement of the tissue relative to the recording sites (drift) and loss of signal from some neurons. We used several methods to confirm whether recorded neurons remained consistent across days, the comparisons of principal component, and the similarity of waveform and interspike interval histograms (ISIH)^77^.

The similarity of waveform (*w*) was defined as the maximum linear correlation coefficient between time-shifted average waveforms^78^, by cross-correlating the waveform as a dot product of time changes. This similarity score is independent of the absolute amplitudes of waveforms and a value of 1 indicates identically shaped spikes. The similarity score (*w*) greater than 0.81 is considered to indicate the same neuron.

The ISIH was fit as a mixture of three log-normal distributions^77^ and described by eight parameters: the mean (μ) and SDs (σ) of the three components and the mixing probabilities of the first two components. The ISIH score (I) comparing the similarity between two sets of ISIH parameters, A and B, is defined as:

$$I\left(A,B\right)=\sqrt{\mathop{\sum }\limits_{i=1}^{8}\frac{{\left({A}_{i}-{B}_{i}\right)}^{2}}{{\sigma }_{i}^{2}}}$$

The difference in parameters between similar ISIHs should be close to zero; A low ISIH score value indicates stability, whereas a high value indicates instability. The ISIH score *I* was converted to the approximately Gaussian I′ by applying the natural logarithm

$$I^{\prime} =\log \left(I\right)$$

The *I'* less than 1 is considered to indicate the same neuron.

These metrics were synthesized to assess whether the neuron was the same across days. In Supplementary Fig. S5e, neurons that fulfilled the criteria were included in the analysis (62.6%, 183 of 292 neurons).

### Cross-correlation analysis

To assess the temporal changes in individual behavior and neural dynamics following TMX administration, we used the dynamic velocity of state 2 and state 3 as neural representation data, and snatch duration and retreat events during food competition as behavioral representation data. The temporal relationship between neuronal and behavioral data was evaluated using cross-correlation analysis^79^, which measures the cross-correlation between two-time series as a function of the time shift of one series relative to the other. Here, the two-time series *y*_1*k*_ and *y*_2*k*_ were lagged by *k =* 0, ± 1, ± 2,…, ± length_min_ (*y*_1_|*y*_2_), and cross-covariance, *c*, was calculated for each possible pairing (*y*_1*t*_,*y*_2*t*_),(*y*_1*t*2_,*y*_2*t*2_), … and so on by

$${c}_{{y}_{1}{y}_{2}}\left(k\right)=\left\{\begin{array}{cc}\frac{1}{T}\mathop{\sum }\limits_{t=1}^{T-k}\left({y}_{1t}-{\bar{y}}_{2}\right) & {\text{if}}\,{k}\,{\ge}\,{0}\\ \frac{1}{T}\mathop{\sum }\limits_{t=1}^{T-k}\left({y}_{2t}-{\bar{y}}_{1}\right) & {\text{if}}\,{k}\, {<}\, {0}\end{array}\right.$$

Here, *T* = length_min_(*y*_1_|*y*_2_) and $\bar{y}$ is the sample mean of the series across time. Cross-correlation was in turn calculated for each cross-covariance value

$${r}_{{y}_{1}{y}_{2}}\left(k\right)={c}_{{y}_{1}{y}_{2}}\left(k\right)\times {\left({e}_{{y}_{1}}{e}_{{y}_{2}}\right)}^{-1}$$

where ${e}_{{y}_{1}}$ and ${e}_{{y}_{2}}$ are the square root of variance. Optimal lag time was based on *k* with the greatest cross-correlation estimate. Time lags were calculated in 1-day intervals. Significance of cross-correlation was determined by permutation test (*n* = 1000; *P* < 0.01).

### Food competition analysis

In the food competition task, the first minute is recorded as a social interaction phase in which two mice freely explore the field without food. After 1 min, a food pellet appears in the center of the field, and the first mouse to pick it up or hold it is considered the defender, another one is considered the challenger. When the challenger mouse attacks the food pellet and successfully removes it from the defender mouse, while keeping the pellet away from the opponent mouse for 3 s, it becomes the new defender. If the challenger mouse fails to remove the donut or maintain it away from the opponent mouse for 3 s during the attack phase, it is recorded as a competition failure. The cumulative occupancy time is defined as the total duration to become the defender.

To more closely observe the behavioral patterns of food competition between pairs of mice, we turned to deep learning multi-animal tracking. Using the SLEAP^15^, we trained a convolutional neural network to perform predictions. We randomly selected and labeled 3000 unlabeled frames of social interaction from 10 videos, with 1000 frames annotated by two individuals to determine human-level accuracy. We used different training-testing ratios to generate non-overlapping sequences of data for multiple rounds of cross-validation.

We quantified the mean accuracy, root mean squared error, and compared them to the accuracy and error of two individuals. In this case, the root mean squared error was defined as the Euclidean distance in pixels between the true position and the network’s predicted position. For each instance in a frame, the Euclidean distance was calculated for each target point (head, left ear, right ear, tail) and the root mean squared error for the same video’s evaluation frames was then averaged to assess tracking quality.

The detection precision of model was defined as

$${Precision}=\frac{{TP}}{{TP}+{FP}}$$

True Positives (TP): the number of positive cases correctly detected.

False Positives (FP): the number of positive cases incorrectly detected.

A detection result is considered correct (i.e., a true positive TP) if it meets the following criteria:

Correct Category: the detected instance's category must match the actual category.

Accurate Localization: the overlap between the bounding box of the detected instance and the true instance's bounding box must exceed a certain threshold, commonly measured by the Intersection over Union (IoU).

The IoU is calculated as follows:

$${IoU}=\frac{{\text{area}}\,{\text{of}}\,{\text{overlap}}\,{\text{between}}\,{\text{the}}\,{\text{predicted}}\,{\text{box}}\,{\text{and}}\,{\text{the}}\,{\text{ground}}\,{\text{truth}}\,{\text{box}}}{{\text{area}}\,{\text{of}}\,{\text{union}}\,{\text{between}}\,{\text{the}}\,{\text{predicted}}\,{\text{box}}\,{\text{and}}\,{\text{the}}\, {\text{ground}}\,{\text{truth}}\,{\text{box}}}$$

if the IoU exceeds a predetermined threshold (60%), the detection is considered correct.

After training, we used SLEAP to track the videos and generate spatial coordinates for each mouse, which were further analyzed using MATLAB.

### Unsupervised clustering for behavioral motifs

The output from SLEAP was used to compute normalized behavioral motif clustering features, for which we used hierarchical clustering to generate a dendrogram and uniform manifold approximation and projection to produce a manifold. The features compose two parts: one part includes the individual’s own physical characteristics, and the other part includes the interaction features between the two mice. The self-features include: length of head, length of body, angle between head and body, ear-to-nose length for each ear, ear-body angle for each ear, the angel and distance displacement of the mouse. The interaction features include: body-to-body angle, head-to-head angel, nose-to-nose distance and angles between mice, distance and angle between the tail of one mouse and the nose of the other. All angles, distances are reported using polar coordinates, the unit of distance and length was pixels. Hierarchical clustering is a bottom-up technique for building clusters based on similarity between data points without specifying the number of clusters, and the results are displayed in a dendrogram. In our case, the clustering was based on the Euclidean distance between combinations of behavioral features extracted in 500 ms clips. These data processing techniques are as follows. First, the data were divided into 500 ms segments. For each segment, we extracted the average individual features from each mouse and the average social features between the two mice. These features were then z-score normalized, and hierarchical clustering was performed using Euclidean distance with the default parameters in MATLAB R2020.

### Electrophysiology data analysis

#### Firing rate analysis and hierarchical clustering for neural activity

To determine whether the neuron had a significant response to event, we used the Wilcoxon sign-rank test to compare the firing rates during a 3 s window starting at the baseline period or onset of the event. If there was a significant change in firing rate, we determined the neuron’s responsiveness to the event based on the average z-score during a 3 s response window. The firing rates were smoothed before calculating the z-score based on the average firing rate during the response window. We arranged the responsiveness of neurons to specific events in a matrix, where columns represented time and rows represented neurons. We used a hierarchical clustering method based on the Euclidean distance and a threshold of 0.4 to determine functional clusters, excluding cells with firing rates lower than 0.1 Hz. We presented neurons with strong responses to events, defined as those with firing rates during event greater than 1.5 z-score or less than or equal to −1 z-score.

#### Dynamical system models of neural data

We employ a rSLDS to model neural activity per mouse individually, following established methods^17^. A rSLDS is a generalization of a Switching Linear Dynamical System (SLDS) in which the switches in the discrete state are allowed to depend on the value of the continuous state (hence the name recurrent). It incorporates three variable sets: discrete states (*z*), continuous latent factors (*x*) that capture the low-dimensional characteristics of neural activity, and the activity of recorded neurons (*y*). The activity of neurons uses firing rates instead of z-scores, without smoothing. The time-bin is set to 500 ms, consistent with the behavior analyses, to facilitate subsequent analysis. The electrical records from all trials of this mouse were integrated and modeled together, the electrophysiological time series data of each trial were truncated to the length of the shortest trial to maintain size consistency.

The generative model for rSLDS is the same as the SLDS case, except that the discrete state transition probabilities are modulated by the continuous state.

1. Discrete state update. At each time step, sample a new discrete state ${z}_{t}{\rm{| }}{z}_{t-1},{x}_{t-1}$ with probabilities driven by a logistic regression on the continuous state:
  1 $$p\left({z}_{t}=i{\rm{| }}{z}_{t-1}=j,{x}_{t-1}\right)\propto \exp \left(\log \left({P}_{j,i}\right)+{W}_{i}^{T}{u}_{t}+{R}_{i}^{T}{x}_{t-1}\right)$$
  where *W*_*i*_ is a vector of weights associated with discrete state *i*, which controls dependence on an external known input *u*_*t*_. Here we do not add external input, so define *u*_*t*_ as a zero matrix. *R*_*i*_ is again a vector of weights assocaited with state *i*, which weights the contribution from the prior state.
2. Continuous state update. Update the state using the dynamics matrix corresponding to the new discrete state:
  2 $${x}_{t}={A}_{k}{x}_{t-1}+{V}_{k}{u}_{t}+{b}_{k}+{w}_{t}$$
  *A*_*k*_ is the dynamics matrix corresponding to discrete state *k*. *u*_*t*_ is the input vector and *V*_*k*_ is the corresponding control matrix. The vector *b* is an offset vector, which can drive the dynamics in a particular direction. The terms *w*_*t*_ is a noise terms, which perturbs the dynamics, modeled as zero-mean multivariate Gaussians.
3. Emission. The observation of the state, according to the specified observation model. The state controls the observation via a Generalized Linear Model:

3 $${y}_{t}{\mathscr{\sim }}{\mathscr{P}}\left(\eta \left({C}_{k}{x}_{t}+{d}_{k}+{F}_{k}{u}_{t}+{v}_{t}\right)\right)$$

*P* is a probabibility distribution. The inner arguments form an affine measurement of the state, which is then passed through the inverse link function $\eta \left(\cdot \right)$. In this case, *C*_*k*_ is the measurement matrix corresponding to discrete state *k*, *d*_*k*_ is an offset or bias term corresponding to discrete state *k*, *F*_*k*_ is called the feedthrough matrix or passthrough matrix (it passes the input directly to the emission). In the Gaussian case, the emission can simply be written as ${y}_{t}={C}_{k}{x}_{t}+{d}_{k}+{F}_{k}{u}_{t}+{v}_{t}$ where *v*_*t*_ is a Gaussian r.v.

The aforementioned parameters are inferred through the employment of maximum likelihood, utilizing approximate variational inference techniques, as expounded upon in great detail in Linderman et al. ^17^. The model’s performance is evaluated using the ELBO by 5-fold cross-validation, which measures the Kullback-Leibler divergence between the true posterior and its approximated form.

The code comes from the SSM package: (https://github.com/lindermanlab/ssm).

#### State-specific GLM

This model is developed with reference to previous research^21^ that used a Bernoulli GLM to predict the types of songs in flies during courtship based on the feedback cues. Here, we instead use a set of multinomial GLM to predict which of four types of behavior (other, snatch, chase, and retreat) a mouse will take at an arbitrary moment in time. Before input, the covariant cues were normalized with z-score. For each discrete state, there is a corresponding multinomial GLM. The GLM model was parameterized by a set of four filters {*F*_*i*_}, *i* ∈ {1, 2, 3, 4}, which map the vector of covariant cues to the non-normalized log probability of each behavior. The probability of each behavior given under the model given feedback cue vector *s*_*t*_ can be written as follows:

$$P\left({\rm{behavior}}=i{\rm{| }}{s}_{t}\right)=\frac{\exp \left({F}_{i}\cdot {s}_{t}\right)}{\mathop{\sum }\limits_{j=1}^{4}\exp \left({F}_{j}\cdot {s}_{t}\right)}$$

Here, we set the first filter (‘other’) of all states to an all-zero matrix, since probabilities must be summed to 1. We fit the model via numerical optimization of the log-likelihood function to find its maximum and used a penalty on the sum of squared differences between adjacent coefficients to impose smoothness.

The chance model was defined as the probability of observing each of the four behaviors (other, snatch, chase, retreat) in a given frame, which was calculated from statistics averaged across all of the competition, we denote as ${p}_{\text{Chance}}({behavior})$. Thus, the probability of observing a particular song mode was determined as follows:

$${p}_{\text{Chance}}\left(\text{behavior}\,{\rm{i}}\right)=\frac{{N}_{i}}{N}$$

where *N*_*i*_ is the number of time bins during the competition with behavior *i*, and *N* is the total number of time bins. The likelihood of observed behavior sequences under the Chance models was computed using test dataset.

#### Low dimensional (PCA) representation of dynamical system

As the latent states remain unchanged under linear transformations, we can transform the high-dimensional rSLDS latent space into an equivalent model using a suitable transformation. We opted for PCA as it enables us to describe the dynamics of the system using a small number of dimensions while retaining the overall behavior. We followed the same procedure as Ann Kennedy^16^ to perform this transformation:

1. Obtain latent variables *x*_1_, *x*_2_, …,*x*_*t*_ from the raw neural data *y*_*t*_.
2. Compute a whitening transformation *W* such that *W*_*x*_ has a covariance matrix of the identity matrix, transforming the raw data to data with the same variance.
3. Apply a linear dynamical system transformation ${x}^{\prime} =W{x}_{t}$ to the transformed data with new emission matrix ${C}^{\prime} =C{W}^{-1}$.
4. Perform a singular value decomposition (SVD) on the new emission matrix ${C}^{\prime} ={US}{V}^{T}$ to obtain matrix $P=S{V}^{T}$
5. Compute the final transformed latent variables ${x}^{\prime\prime}$ by multiplying ${P}^{-1}$ with the whitening transformation and the transformed data. ${x}^{\prime\prime} ={P}^{-1}{x}_{t}{\prime} ={P}^{-1}W{x}_{t}$

After final transformation, the first two components of ${x}_{t}^{\prime\prime}$ account for the highest variance in the raw neural data *y*_*t*_, as the singular values are ordered. This PCA-based approach also takes into account the emission matrix C of the fitted linear dynamical system.

#### Dynamic velocity measurement

We followed the same procedure as Ann Kennedy^16^ to calculate the dynamic velocity to measure the average rate of change intrinsically generated by the fitted dynamical system during a specific state of interest. We initially computed the average norm of *A*_*z*_ *x*_*t*_ for each value of *x*_*t*_ linked to a specific state *z*. We calculated the mean of this metric across states, which we define as

$${V}_{z}=\frac{1}{n({T}_{z})}\sum _{t\in {T}_{z}}{{\Vert }}{A}_{z}{x}_{t}{{\Vert }}$$

*Z* represents the set of states, *T*_*z*_ represents the set of all time points during state *z*, ||·|| denotes the Euclidean norm, and *n*(·) denotes the number of elements in a set. *A*_*z*_ represents the dynamic matrix of the discrete state. Finally, we normalized this value relative to the dynamic velocity of state1 to facilitate comparison across animals.

#### Rotational angle calculation

For each individual’s each rotational trajectory in 2D PCA subspace, each time moment *t* corresponds to a specific rotation angle. The rotation angle is determined by three points: (1) The attractor point of state 1 (the point in state 1 that shows the lowest of Euclidean norm of *A*_*z*_*x*_*t*_). (2) The geometric median of the rotational trajectory, calculated by Weiszfeld algorithm. (3) The position of the population vector at time t.

Based on these three points, we can define two vectors: the first vector ***u*** points from (2) to (1), and the second vector ***v*** points from (2) to (3). We calculate the angle between the two vectors based on the dot product, as:

$$\theta ={\cos }^{-1}\left(\frac{{\boldsymbol{u}}\cdot {\boldsymbol{v}}}{\parallel {\boldsymbol{u}}\parallel \parallel {\boldsymbol{v}}\parallel}\right)$$

Here, ***u*** and ***v*** are the two vectors, · denotes the dot product, the ||***u***|| and ||***v***|| represent the magnitudes of the vectors.

Subsequently, we use the 2D cross-product to determine the direction of the rotation. For two-dimensional vectors ***u*** = (*u*_*x*_, *u*_*y*_) and ***v*** = (*v*_*x*_, *v*_*y*_), the cross product is simplified to:

$${\rm{sign}}={u}_{x}{v}_{y}-{u}_{y}{v}_{x}$$

If sign is positive, ***u*** is in the counterclockwise direction relative to ***v***, if sign is negative, ***u*** is in the clockwise direction relative to ***v***.

Then based on the sign of the cross product, we adjusted the initially computed angle:

- If the rotation direction is consistent with the direction of the trajectory’s rotation, use *θ* as the final angle.
- If the rotation direction is not consistent with the direction of the trajectory’s rotation, use (360^o^ – *θ*) as the final angle to reflect clockwise rotation.

#### Calculation of attractor score and stability

We calculated the magnitude of the largest time constant as the attractor stability score for the given state, and the time constant for each mode of the linear dynamical system was calculated by the eigenvalues (λ) of the system’s dynamics matrix, as previously derived by Maheswaranathan^80^ et al. as:

$${\tau }_{a}=\left|\frac{1}{\log \left(\left|{\lambda }_{a}\right|\right)}\right|$$

And the line attractor score defined as:

$${\rm{line\; attractor\; score}}={lo}{g}_{2}\frac{{t}_{n}}{{t}_{n-1}}$$

*t*_*n*_ is the largest time constant of the dynamics matrix of a dynamical system and ${t}_{n-1}$ is the second largest time constant. This score would be close to zero in the absence of line attractor dynamics. Conversely, it would be greater than one for systems with a line attractor.

### Statistical analysis

The data are expressed as the means ± SEM. Statistical analyses were performed with GraphPad Prism 6 (GraphPad Software, San Diego, CA, USA). Statistical differences between two groups were analyzed using independent sample *t*-tests, and two-way analysis of variance (ANOVA) was used to compare the differences among multiple groups. Correlations were assessed by linear regression and Pearson’s product-moment correlation. *P* < 0.05 was considered to indicate statistical significance.

## Supplementary information

Supplementary figures

Supplementary Video S1

Supplementary Table S1
