# Supplementary material for: Historical loss weakens competitive behavior by remodeling ventral hippocampal dynamics
Source: Cell Discov. 2025 Feb 25;11:16. doi: 10.1038/s41421-024-00751-3 (PMC11850767; doi:10.1038/s41421-024-00751-3)
Supplement: Supplementary file 1 — Supplementary figures [file 41421_2024_751_MOESM1_ESM.pdf]

Supplementary figure and figure legends

Supplementary Fig. S1

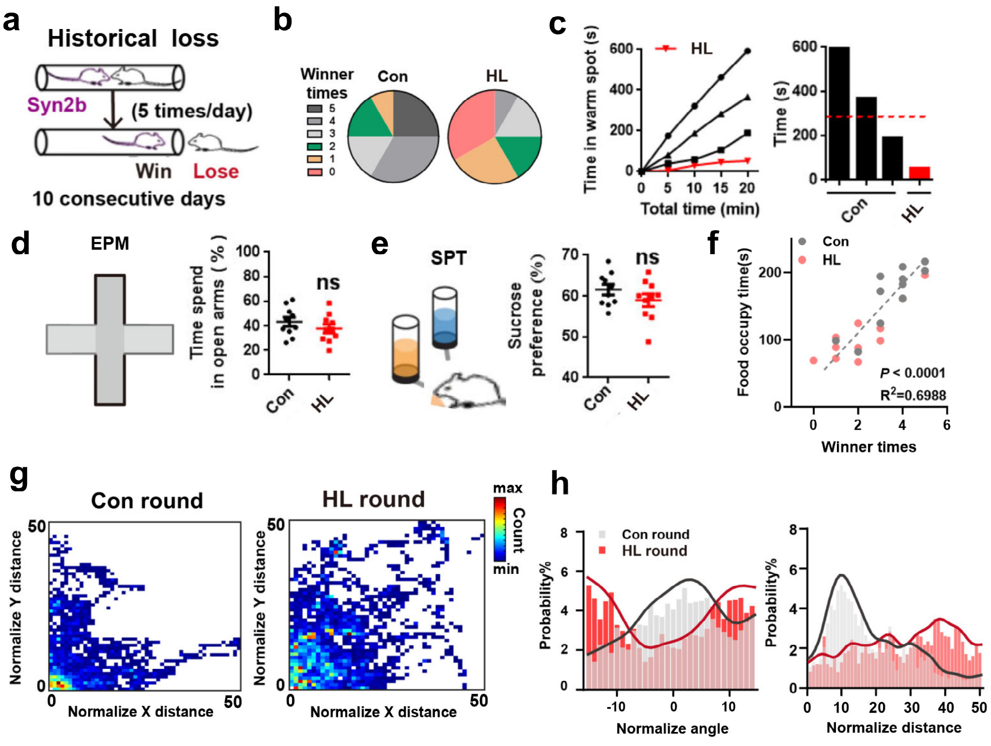

Supplementary Fig. S1| HL mice exhibit stable submissive behaviors. a Schematic diagram of Historical loss. **b** Winner times of HL mice and Con mice in the single-housed tube test. *n* = 11 for each group. Chi-square test, $x^2=15.4$, $P < 0.01$. **c** Left: Cumulative time of each individual mouse in a 20-min warm-spot test. Right: Total time in the warm spot of four group-housed mice. The dashed line indicates the expected time of spot occupation if there were no differences among mice in the warm spot. **d-e** The percentage of the time spent in the open arms (d) in the elevated plus-maze (EPM) test, and the percentage of sucrose consumption (e) in the sucrose preference test (SPT) were evaluated. *n* = 10 for each group. The data are presented as the means ± SEM, Student's t test, ns, no significance. **f** Correlation between food occupancy time in food competition task and winner times in the tube test. Pearson's correlation test, $P < 0.0001$. **g** The heatmap of area distribution occupied by the challenger mice (Con or HL) relative to the defender mouse in a representative food competition trial. The relative XY distance is normalized into 50 bins. **h** Left: Angle distribution of Con/HL mouse as challenger relative to the defender mouse. Right: Distance distribution of Con/HL mouse as challenger relative to the defender mouse. *n* = 11 mice for each group.

Supplementary Fig. S2

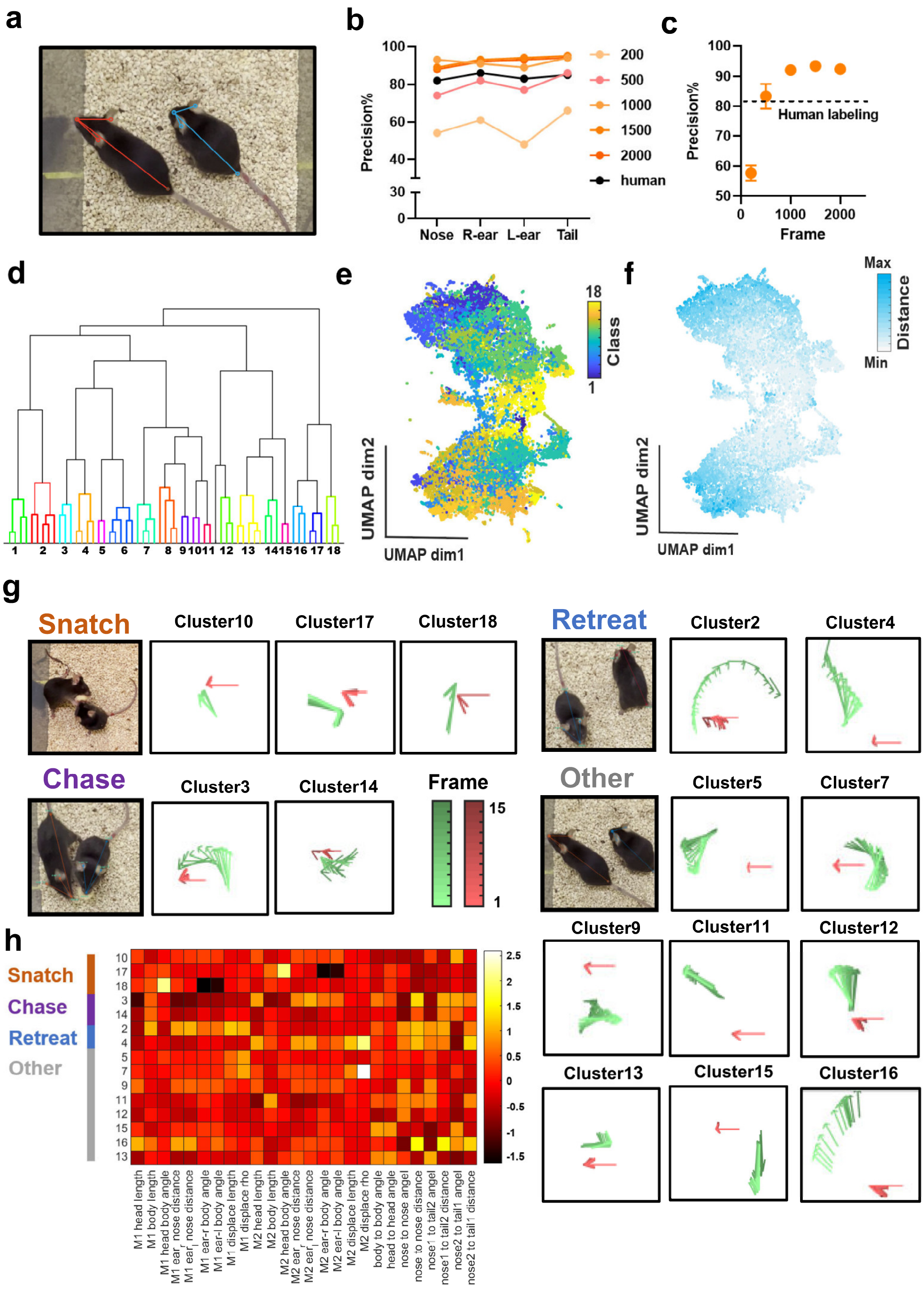

Supplementary Fig. S2| SLEAP tracking metrics and behavior classification.

a Video frame with body-part tracking. **b** Precision of SLEAP tracker for tracking different body parts in high-resolution videos (1920 x 1080 pixels) at training set with different frame numbers. **c** Total Precision at training set with different frame numbers. The performance of the SLEAP tracker surpassed human-level performance when the training frame numbers were more than 500. **d-e** Dendrogram (d) and UMAP visualization (e) depicting all video clips color-coded by cluster ID for clustering of food competition behavior. **f** UMAP embedding from all video clips in food competition behavior color-coded by nose-to-nose distance between mice. **g** Left: example frames from clips of a specific cluster. Right: average normalized skeleton base across clusters for social behavior clustering over a 500 ms video clip containing 15 frames, each arrow represents 1 frame (33.3 ms). Red arrow indicates the defender and green indicates the challenger. **h** Heatmap of normalized values for the individual and interaction features, which were classified in four behavioral classes.

Supplementary Fig. S3

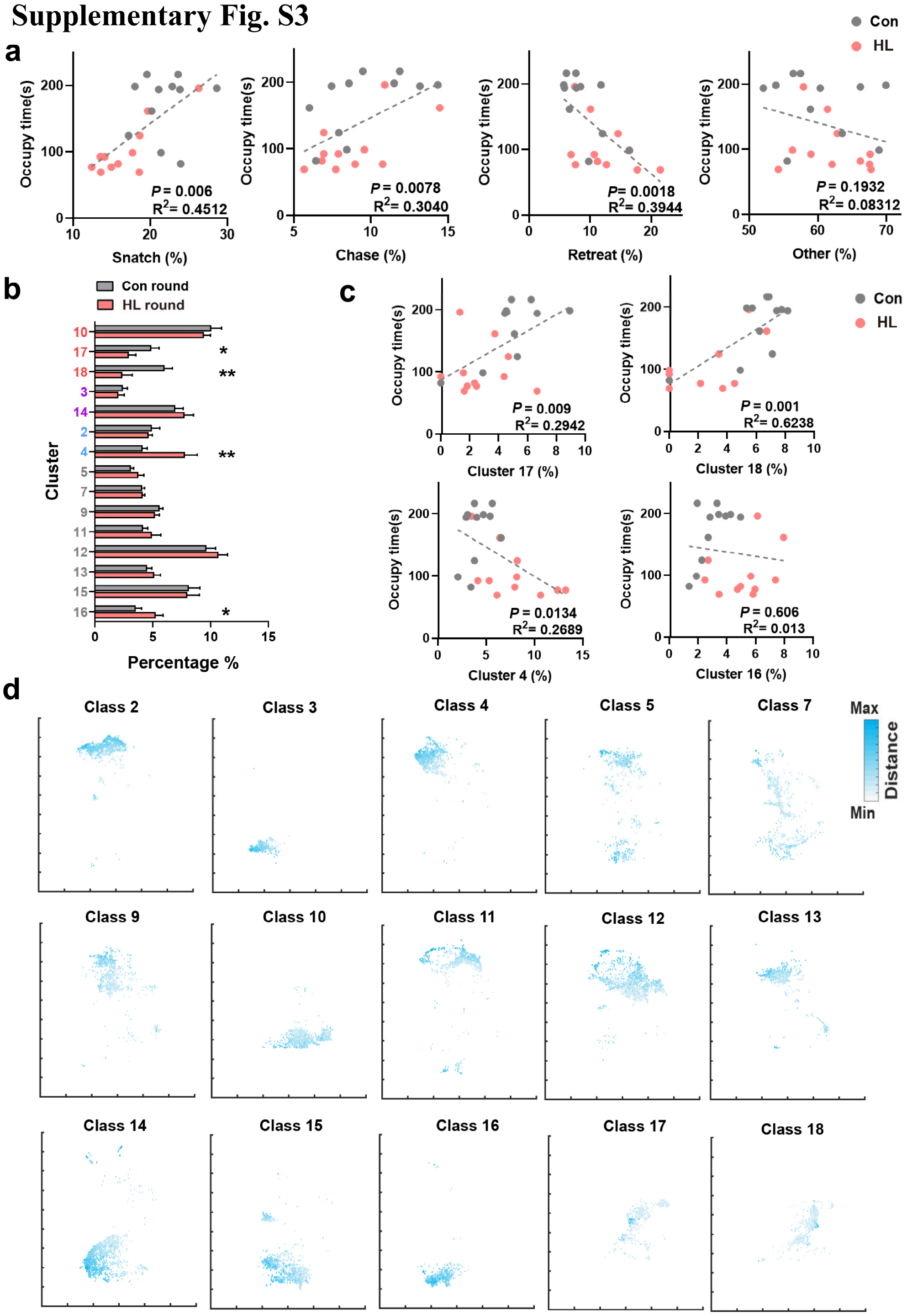

Supplementary Fig. S3| The unsupervised clustering results for behaviors.

a Relationship between frame percentage of specific behavior class during challenger rounds and total food occupancy time for individuals. Chase, $P = 0.0078$; Snatch, $P = 0.006$; Retreat, $P = 0.0018$; Other, $P = 0.1932$; $n = 22$ mice. **b** Percentage of frames that correspond to behavioral cluster out of the total frames in Con round and HL round (**$P < 0.01$, *$P < 0.05$, $n = 11$ mice for each group). **c** Relationship between frame percentage of behavior clusters during challenger rounds and total food occupancy time for individuals. **d** UMAP embedding of each cluster in food competition behavior color-coded by distance between mice. Each scatter represents the average features of 500 ms video clip which contains 15 frames.

Supplementary Fig. S4

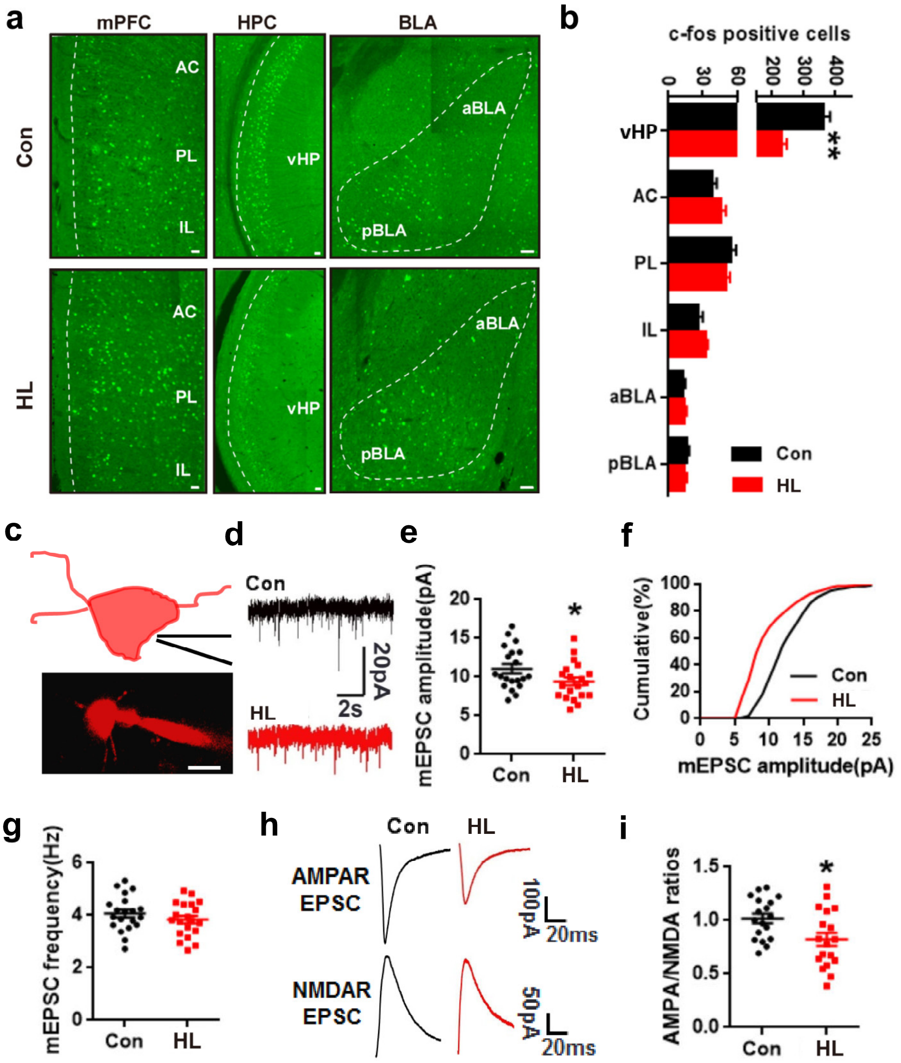

Supplementary Fig. S4| HL results in neuronal inactivation in vHPC.

a Representative immunohistochemical images showing the c-fos-positive neurons in the ventral hippocampus (vHP), mPFC, BLA of HL mice and control mice after food competition task. Scale bars = 200 μm. **b** Quantification analysis of c-fos intensities of the images presented in **a** (**$P < 0.01$, $n = 8$ mice for each group). **c** Representative images of ventral hippocampal neurons in the patch clamp. **d** Representative traces of mEPSCs in ventral hippocampal neurons in HL and Con mice. **e-g** The mean mEPSCs amplitudes (e) and representative cumulative distribution of the mEPSCs amplitudes (f) and frequencies (g) were analyzed; $n = 20$ neurons from four mice of each group. **h** Representative traces of NMDA receptor (NMDAR)- and AMPA receptor (AMPAR)-mediated currents recorded from ventral hippocampal neurons of HL and Con mice, respectively. **i** The AMPA/NMDA ratios in Con and HL mice, respectively. $n = 20$ for each group. The data are presented as means ± SEM, Student's t test. * $P < 0.05$.

Supplementary Fig. S5

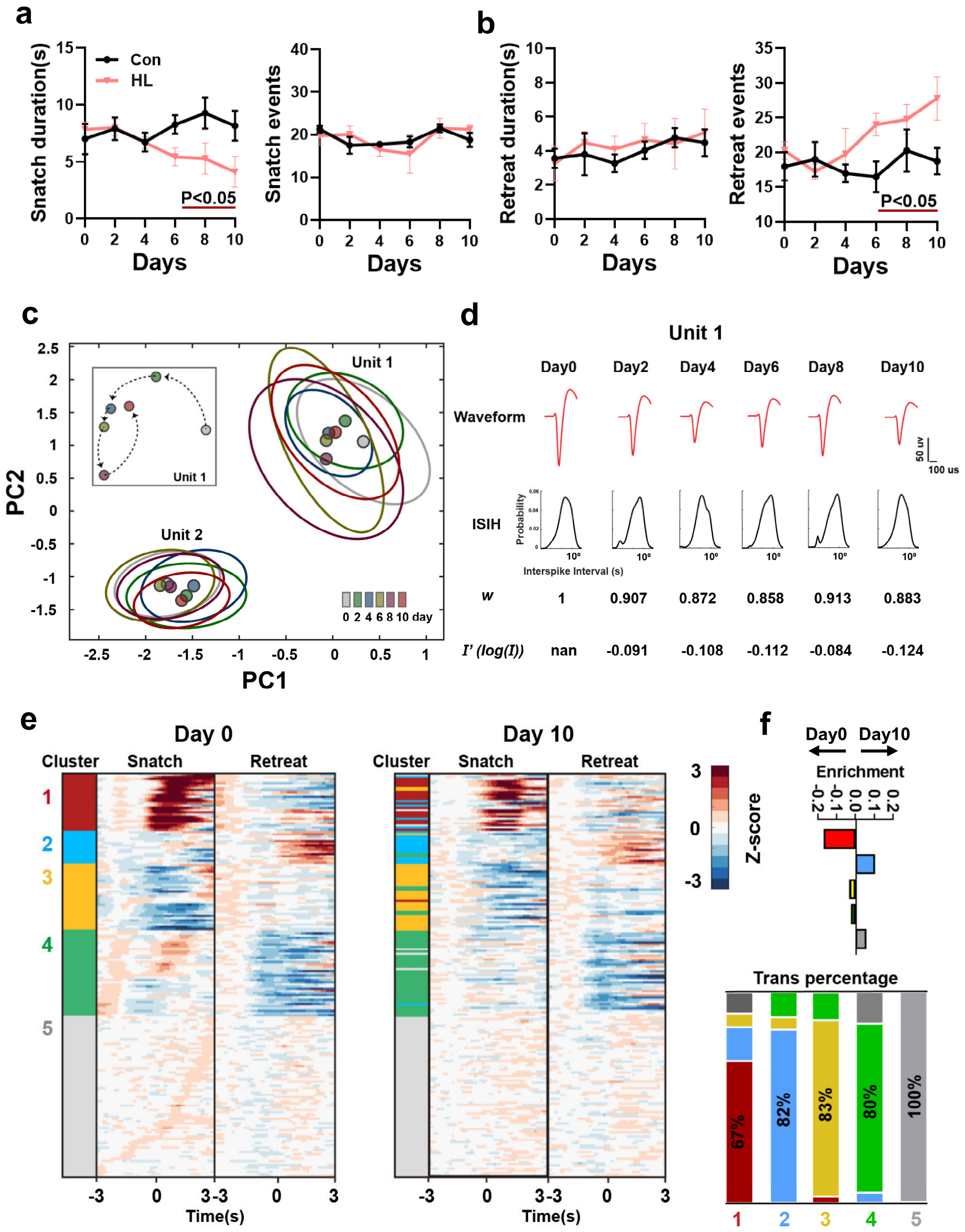

Supplementary Fig. S5| The changes of neural activity and competitive behavior during the HL process. a-b Continuous changes in the snatch behavior (a) and retreat behavior (b) during food competition for individuals undergoing HL training or Control (n = 4 for each group). **c** Example of principal component (PC) analysis for two units across multiple days. Dots: centroid of the PC distribution. Ovals: 2σ contour of PC distribution. Colors code the time stamps. Inset: The evolution over time of the centroid of the PC for unit 1. **d** The waveforms and ISIH of the unit shown in **c** over the recording across multiple days. w: The waveform similarity score; I: The ISIH similarity score. **e** Heatmap of vHPC neurons responses to two specific behavioral classes in food competition for day 0 (left) and day 10 (right). Colors represent clusters derived from hierarchical clustering. Cell clusters with a Z-score greater than 1.5 or less than -1 were considered responsive to the event (n =120 neurons). Clusters without responsive are labeled in grey. **f** Upper, difference in percentage enrichment of functional cluster cells between day 0 and day 10. Bottom, the distribution of functional cluster transformations after undergoing HL process. Cluster 1 transitions into cluster 1 (67.7%), cluster 2 (16.1%), cluster 3 (6.4%), cluster 4 (0%) and cluster 5 (9.6%); Cluster 2 transitions into cluster 1 (0%), cluster 2 (82.3%), cluster 3 (5.8%), cluster 4 (11.7%) and cluster 5 (0%); Cluster 3 transitions into cluster 1 (3.2%), cluster 2 (0%), cluster 3 (83.8%), cluster 4 (12.9%) and cluster 5 (0%); Cluster 4 transitions into cluster 1 (0%), cluster 2 (4.8%), cluster 3 (0%), cluster 4 (80.5%) and cluster 5 (14.6%); Cluster 5 transitions into cluster 1 (0%), cluster 2 (0%), cluster 3 (0%), cluster 4 (0%) and cluster 5 (100%). Colors correspond to the heatmap.

Supplementary Fig. S6

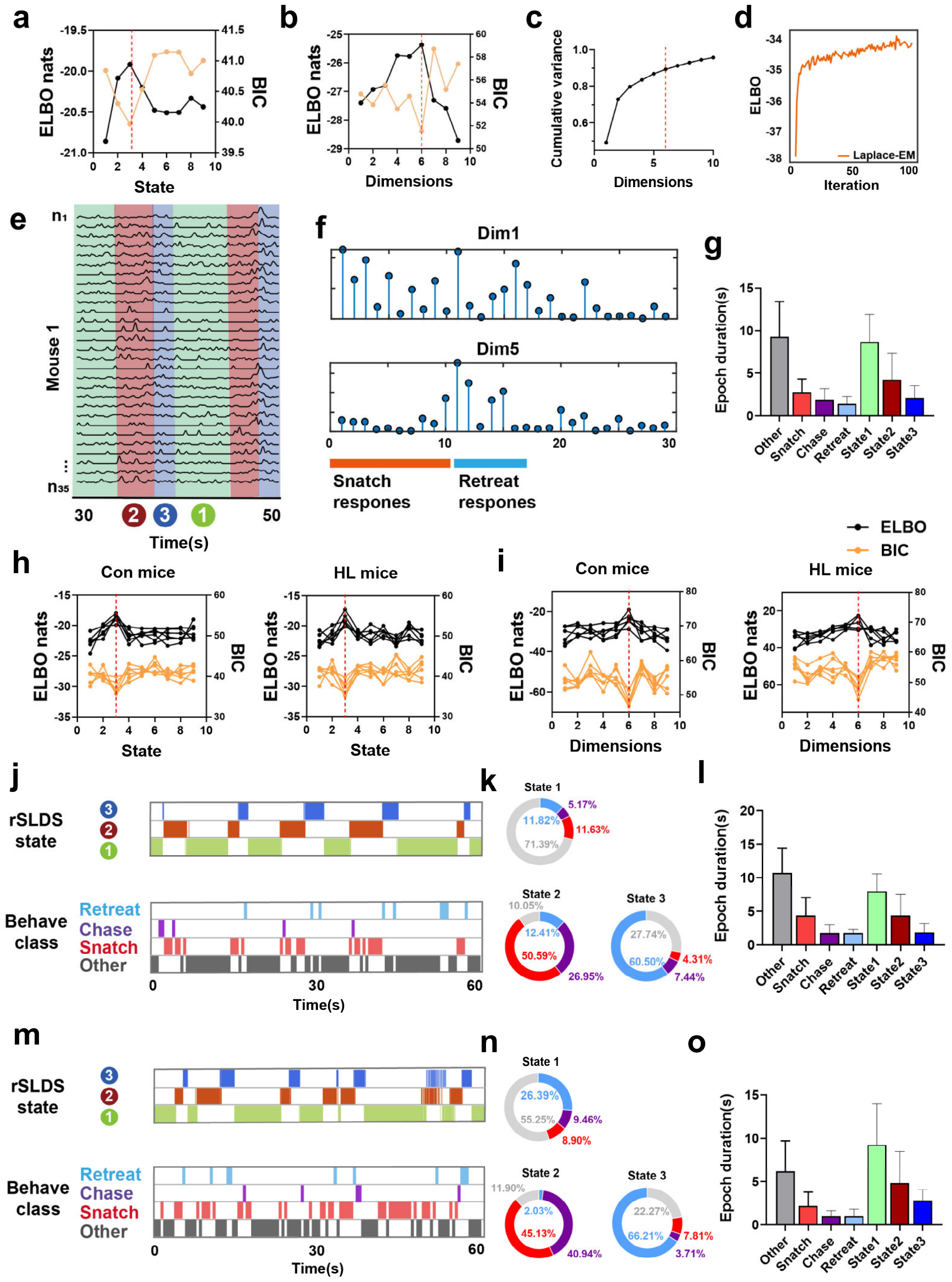

Supplementary Fig. S6| The rSLDS model for vHPC population activity.

a Optimization of the number of rSLDS discrete states in HL mouse 1. Model performance is measured as Evidence Lower Bound (ELBO) and Bayesian Information Criterion (BIC). **b** Same as (a), but for dimensionality. **c** Variance explained by the dimension chosen in **b**. **d** Convergence of the model performance. **e** A rSLDS state example clip of HL mouse 1 that captured from the (Fig. 2a) at 30s to 50s, n represents neuron. **f** Absolute rSLDS weight of vHPC neurons in HL mouse 1 to integration dimensions, neurons sorted based on their responsiveness to the behavioral events. **g** Timescale of behavior durations and states epochs of HL mouse 1. **h** The ELBO and BIC of rSLDS model under different hyperparameter (states) in Con (left) and HL mice (right). **i** The ELBO and BIC of rSLDS model under different hyperparameter (dimensions) in Con (left) and HL mice (right). **j** A comparison of rSLDS states with annotations of food competition behaviors for Con mouse 1. **k** Composition of behaviors within rSLDS discrete states for Con mouse 1. **l** Timescale of behavior durations and states epochs of Con mouse 1. **m-o** As same as **j-l** but for HL mouse 2.

Supplementary Fig. S7

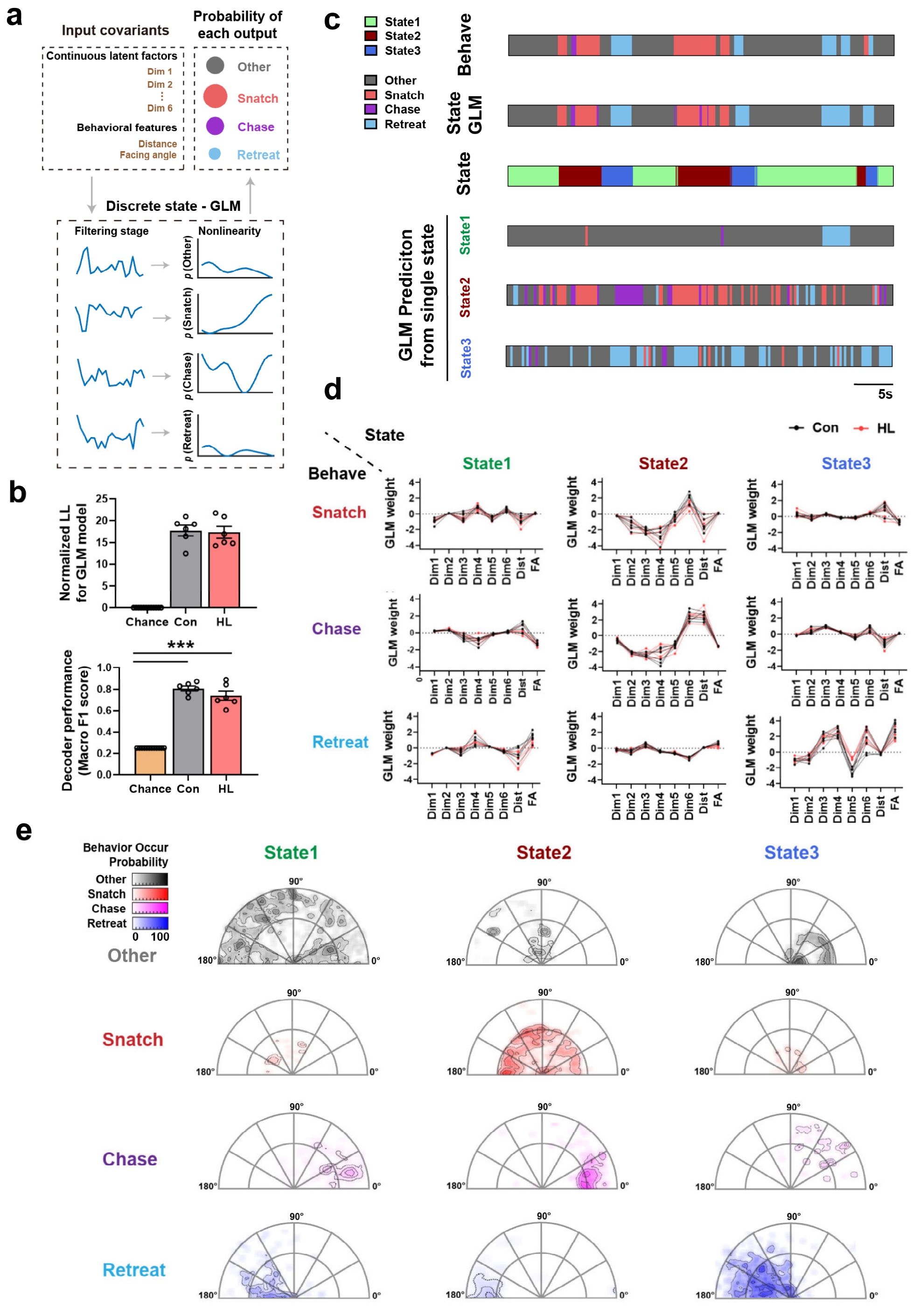

Supplementary Fig. S7| Distinct mappings between continuous latent variable and behaviors under discrete internal states. **a** Diagram of state-specific generalized linear model (State-GLM) data pipeline to decode behavioral class outcome based on latent factors data for the rSLDS model and behavioral feature. **b** (Upper) Normalized log-likelihood (LL) for model on data based on Con or HL mice, chance level as baseline. (Bottom) For each mouse individually, the performance (average Macro F1 score) of model in decoding behavior. **c** An example segment of behavior prediction by the State-GLM model: first row shows the individual's actual behavior during food competition segment; second row presents the behavior prediction by the State-GLM; third row shows the state occupancy distribution inferred by rSLDS; fourth to sixth rows show behavior predictions using GLM filters from single states only. **d** Inferred GLM weights for each mouse, for each of the three behaviors in each three states. **e** Heatmaps of the probability distribution of behavior occurrence in different states, based on the facing angles and distances to their opponents (n = 12 mice). The polar angle of points corresponds to the facing angle, and the radial distance corresponds to the normalized distance. The facing angle of 180-degree represents the mice face-to-face, and the 0 radial distance represents the minimum distance between mice. The dashed lines represent probability distribution contour lines.

Supplementary Fig. S8

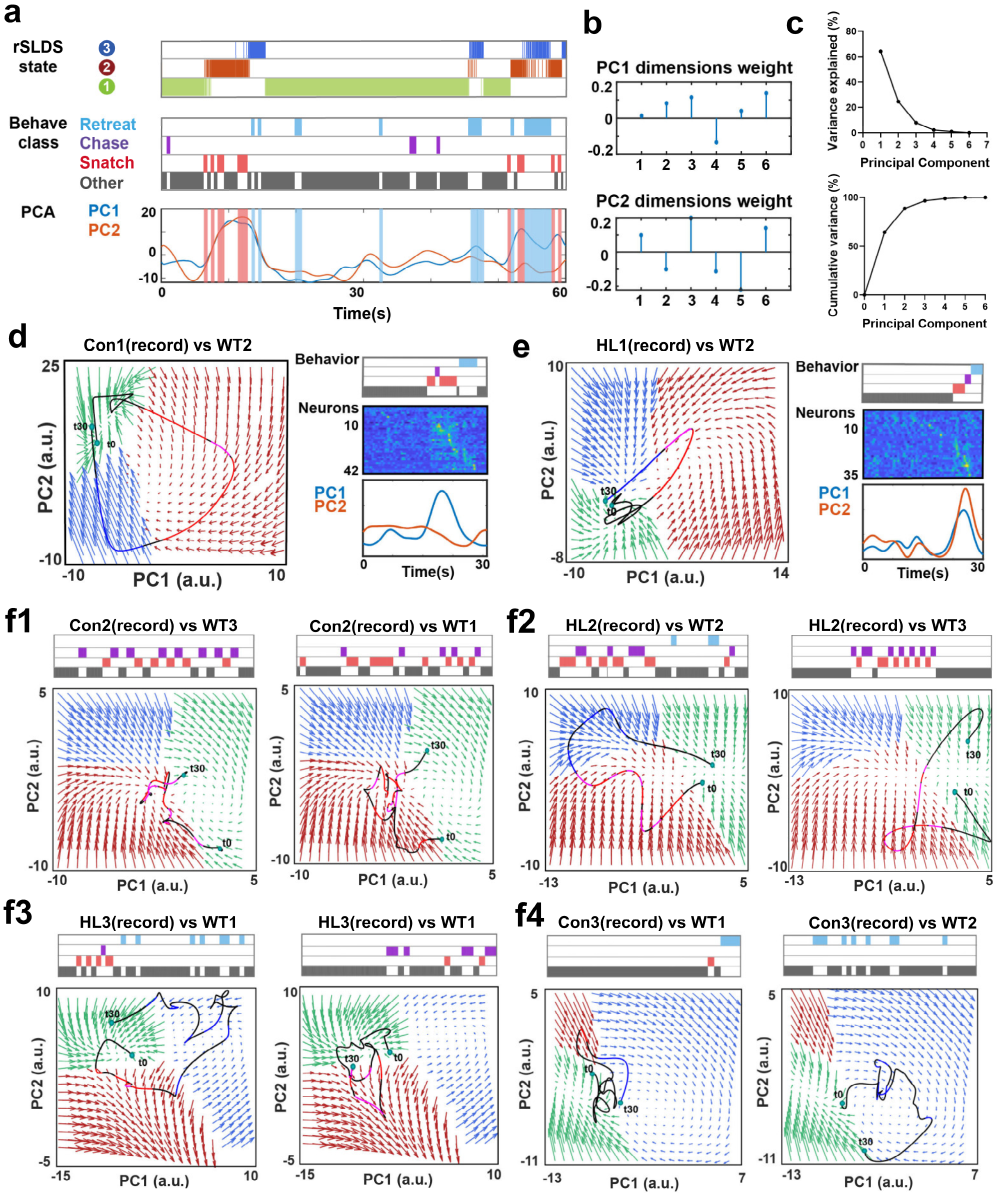

Supplementary Fig. S8| Dimensionality reduction of latent factors by PCA.

a A comparison between rSLDS states, annotations of food competition behavioral class, and snatch/retreat behavior rasters shown with first two principal components of latent factors. **b** PCA weights of PC1 (top) and PC2 (bottom) on latent factors of dynamical system in Con mouse 1. **c** Variance explained by PCA dimension. **d-e** The representative rotational trajectory for competition episodes in Con mouse 1(d) and HL mouse 1(e), as same as Fig. 3d, e. **f1-f4** Same as **d**, for Con mouse 2, 3 and HL mouse 2, 3.

Supplementary Fig. S9

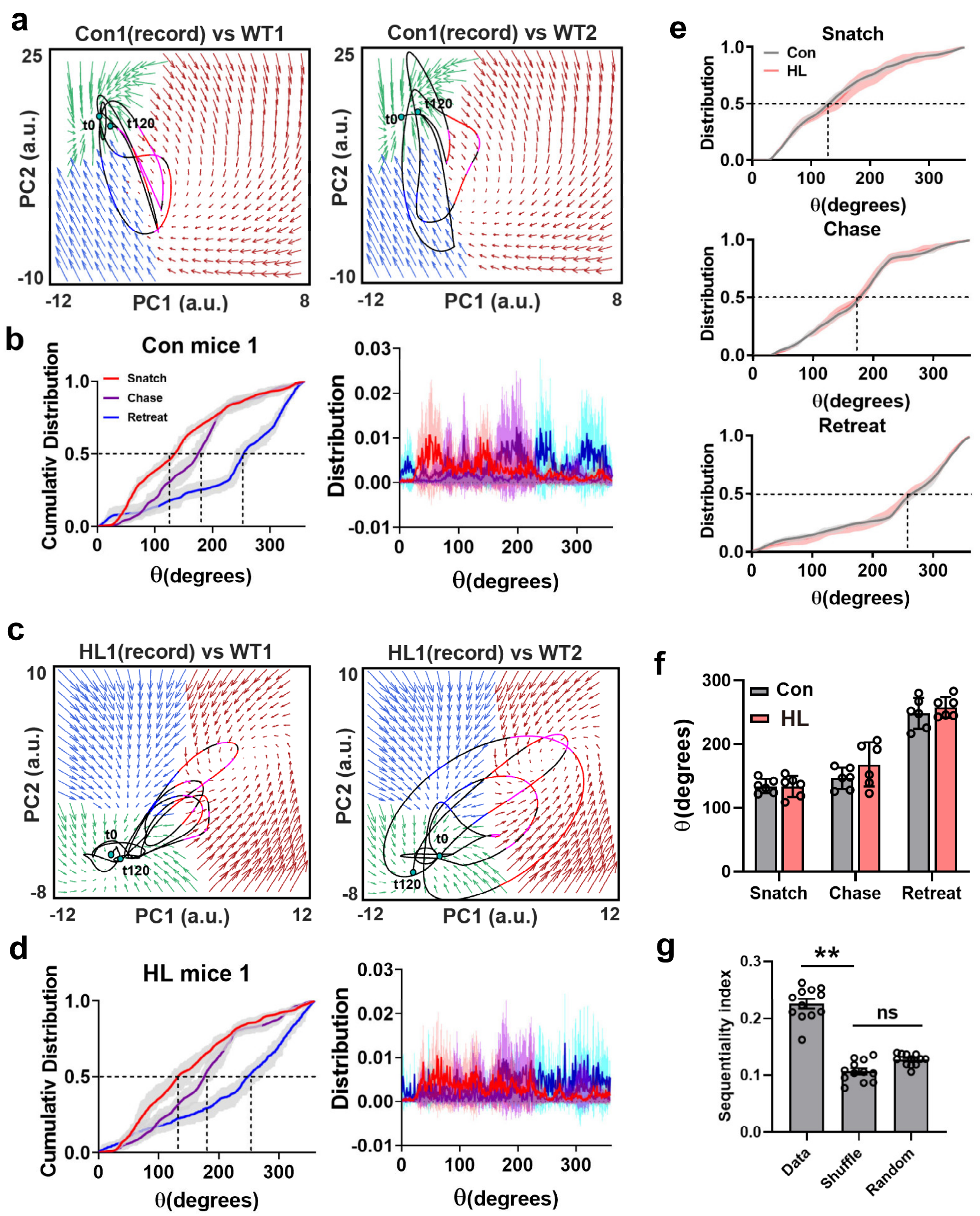

Supplementary Fig. S9 | Con mice and HL mice exhibit the same distribution between rotational angle and behaviors occurrence. a Example segment of rotational dynamics in Con mouse 1 during different competition experiments. **b** (Left) The corresponding empirical cumulative distribution of rotation angle (**θ**) for three behavioral class from Con mouse 1 (*n* = 42 trials). (Right) The probability density distribution of rotation angle (**θ**) for three behavioral class from Con mouse 1. **c** the same as **a**, but for HL mouse 1. **d** the same as **b**, but for HL mouse 1(*n* = 38 trials). **e** Empirical cumulative distribution of rotation angle (**θ**) for three behavioral class in Con or HL mice (*n* = 6 mice for each group). **f** Quantification of 50% distribution point of rotation angle (**θ**) for various behavioral classes in Con mice or HL mice. **g** Sequentiality index for vHPC neural data (Student's t test, ***P* < 0.01, *n* = 12 mice).

Supplementary Fig. S10

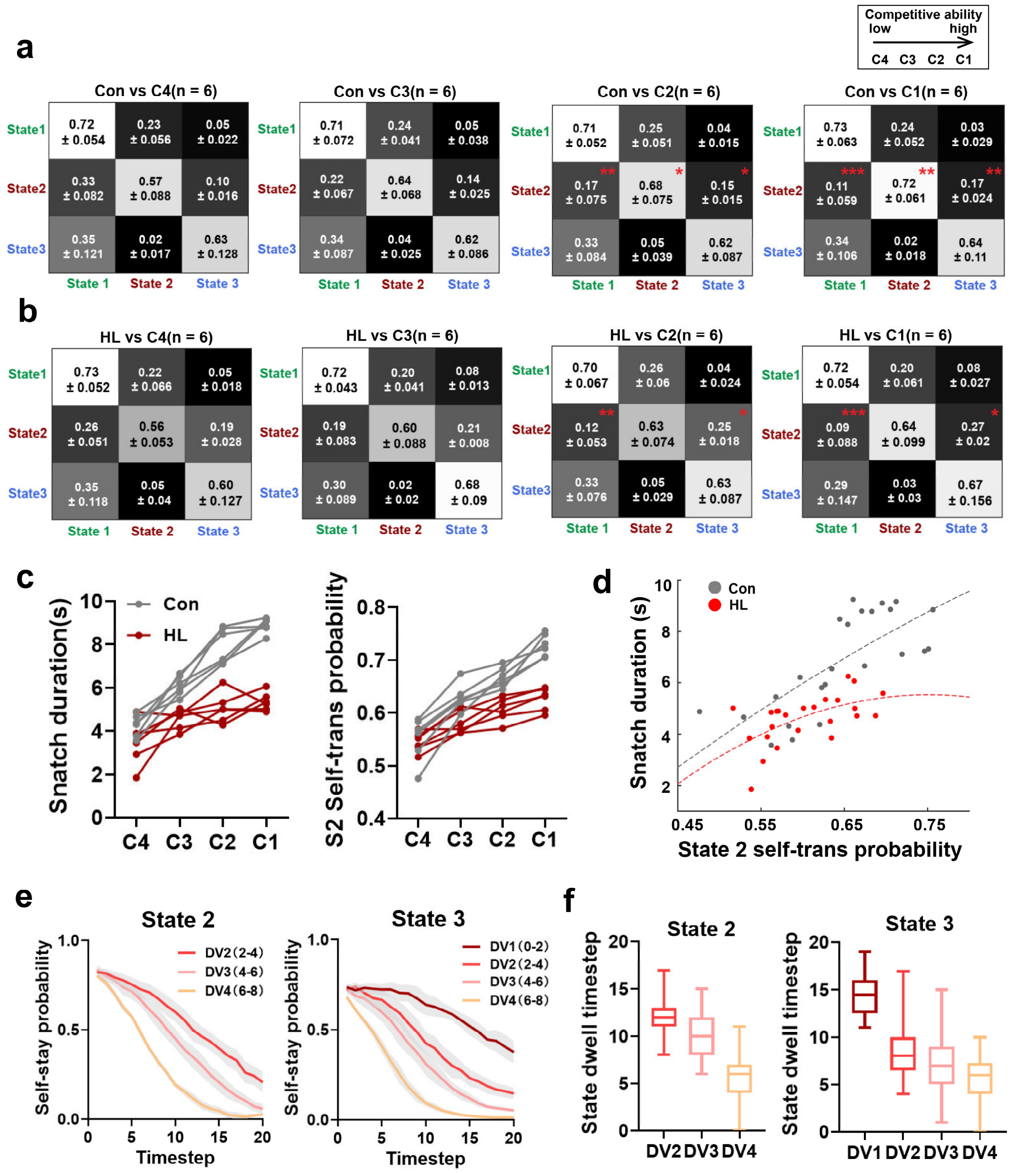

Supplementary Fig. S10| Discrete state transitions in vHPC dynamics of individuals under competition with varying levels of competitiveness.

a Retrospective discrete state transition matrix of Con mice when facing opponents with four different levels (high to low: C1 to C4) of competitiveness. The numbers represent the mean ± std. Student's t test. * $P < 0.05$, ** $P < 0.01$, *** $P < 0.001$. **b** the same as **a**, but for HL mice. **c** Snatch duration (Left) and S2-S2 self-trans probability (Right) of Con or HL mice when facing opponents with four different levels of competitiveness. $n = 6$ mice for each group. **d** Scatter plot of snatch duration and state 2 self-transition probability, with each point representing a single competition. The dashed line represents the fitted binomial curve. **e** The probability of self-stay in the state 2 (left) or state 3 (right) over time steps at different dynamic velocity (high to low: DV4 to DV1). **f** The average dwell timestep in a state 2 (left) or state 3 (right) at different dynamic velocity. State2: DV2 ($n = 27$ trials); DV3 ($n = 43$ trials); DV4 ($n = 111$ trials); State3: DV1 ($n = 16$ trials); DV2 ($n = 49$ trials); DV3 ($n = 74$ trials); DV4 ($n = 46$ trials).

Supplementary Fig. S11

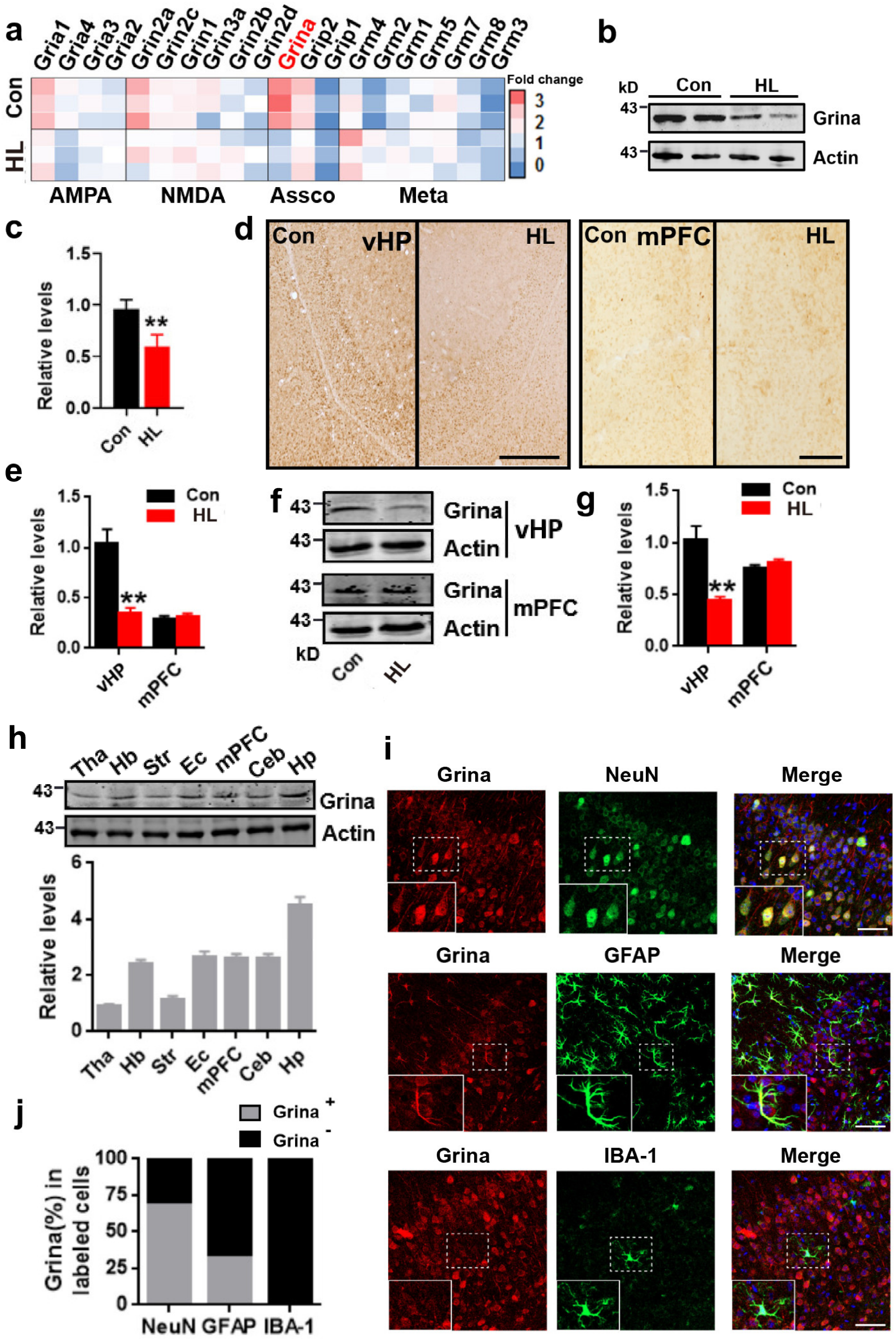

Supplementary Fig. S11| HL results in a reduced *Grina* expression in the hippocampus. a Heatmap illustrating the HL-induced changes of glutamate receptor-related genes in the ventral hippocampus of HL mice and control mice. Assco: glutamate receptor-associated genes; Meta: metabotropic glutamate receptor genes. The color of the heatmap represents the fold change as indicated in the right side. **b** Representative blots of *Grina* protein levels in the ventral hippocampus of Con mice and HL mice. Actin was used as the loading control. **c** The quantified protein levels were quantified between HL mice and control mice in ventral hippocampus. ** $P < 0.01$(ANOVAs). **d** Representative immunohistochemical images showing the expression levels of endogenous *Grina* in regions of the vHPC and mPFC in HL mice and control mice. Scale bars = 200 µm. **e** The anti-*Grina* intensities in the images shown in **d** were quantified, ** $P < 0.01$ (ANOVAs). **f-g** Representative blots of *Grina* protein levels in regions in the vHPC and mPFC of HL mice and control mice (f); and the quantified protein levels (g). $n = 3$ for each group; **$P < 0.01$ (ANOVAs). **h** The protein levels of *Grina* in the thalamus (Tha), hindbrain (Hb), striatum (Str), entorhinal cortex (Ec), medial prefrontal cortex (mPFC), cerebellum (Ceb), and hippocampus (Hp) of WT mice. Actin was used as a loading control. **i** Representative images of immunofluorescence double staining of *Grina* and NeuN (upper panel), GFAP (middle panel), and IBA-1 (lower panel) in hippocampal sections. DAPI in blue; Scale bars=50 µm. **j** The percentage of *Grina* in labeled cells. Gray indicates *Grina*-positive, while black indicates *Grina*-negative.

Supplementary Fig. S12

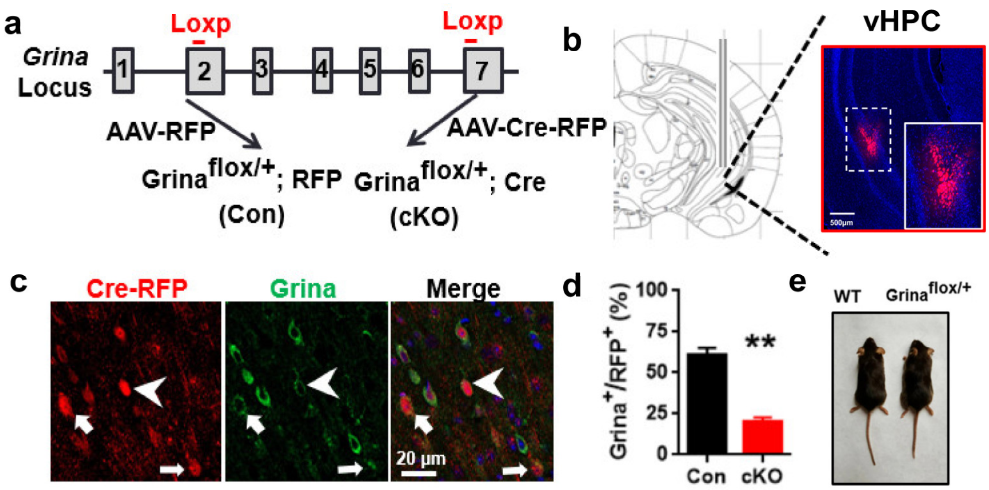

Supplementary Fig. S12| Generation of *Grina* conditional knockout mice.

a A schematic diagram showing the mouse *Grina* gene structure and the strategy to generate the conditional *Grina* knockout mouse by Cre-loxp system. **b** A diagram showing virus injection site (left) and the representative image of virus expression. **c** Representative images of immunofluorescence double staining of *Grina* (green) and Cre-RFP (red) in hippocampal slices. DAPI in blue; Scale bars = 20 μm. **d** The percentage of *Grina* in virus labeled cells (c) in Con or cKO group. $P < 0.01$, $n = 3$ mice for each group. **e** Representative images of body sizes with different genotypes at 3-month–old.

Supplementary Fig. S13

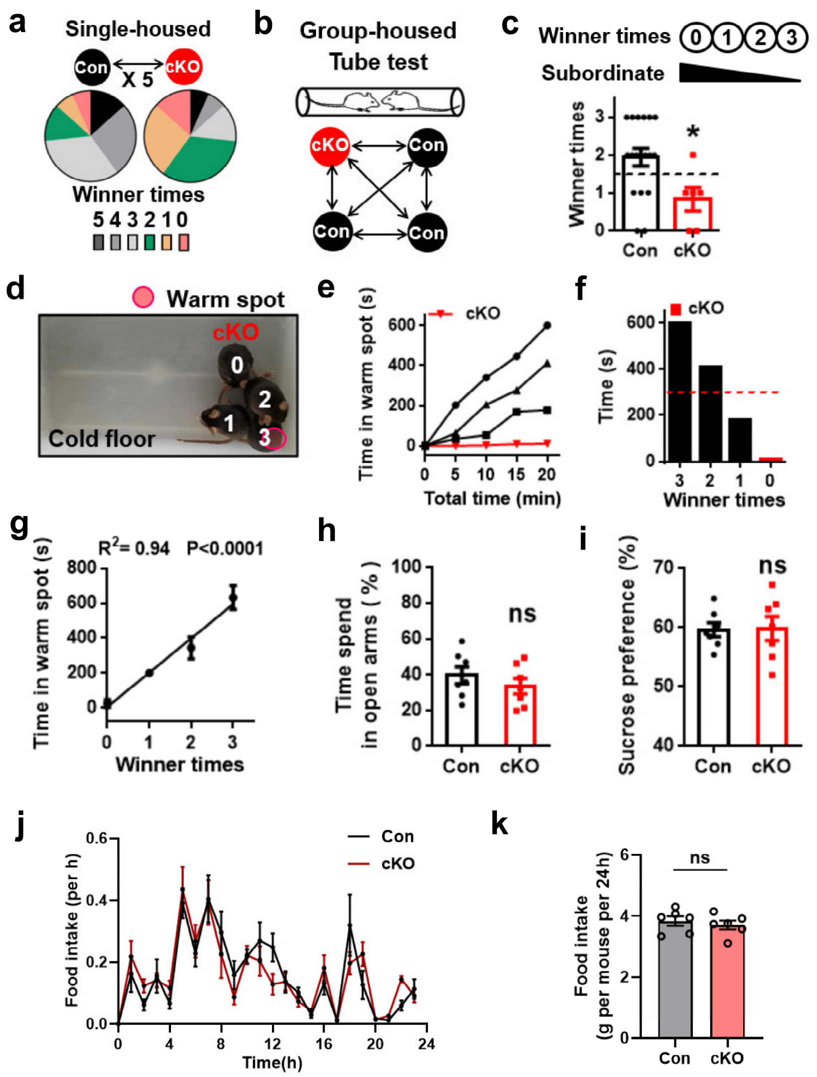

Supplementary Fig. S13| Genetic ablation of *Grina* in vHPC leads to a lower social competitiveness without affecting memory and emotion. a Single-housed control (Con) and *Grina* conditional knockout (cKO) mice with no prior experience with each other were subjected to a tube test, and their winner times in five trials were calculated. $n = 8$ for each group. Chi-square test, $x^2=13.7$, $P < 0.01$. **b** Schematic illustration of the group-housed tube test used to measure social hierarchy. One *Grina* cKO mouse and three Con mice were group housed for 2 weeks and then subjected to tube test. **c** Winner times in the group-housed tube test of each individual mouse in six cages. The dashed line indicates the expected winner times if no differences exist between *Grina*-cKO and Con mice in the tube test. **d** Schematic diagram of the warm spot test, the numbers represent the winner times in the group-housed tube test. **e** Cumulative time of each individual mouse in 20-min warm spot test. **f** Total time in the warm spot of four cage mate mice of different winner times in the tube test. The dashed line indicates the expected occupied time if there were no differences among each mouse in the warm spot. **g** Correlation between the time in the warm spot and winner times in the group-housed tube test. Pearson's correlation test, $P < 0.0001$. **h-i** Both *Grina* cKO and Con mice were subjected to the elevated plus-maze (EPM) and sucrose preference test (SPT) at 3 months. The percentage of the time spent in the open arms of the EPM (h) and the percentage of sucrose consumption in the SPT (i), $n = 6$-8 per group. Data are presented as means ± SEM, Student's t test, ns, no significance. **j** 24-hour food intake monitoring for Con and cKO mice. **k** Average food intake of Con and cKO mice during 24-hour monitoring ($n = 6$ mice each group).

Supplementary Fig. S14

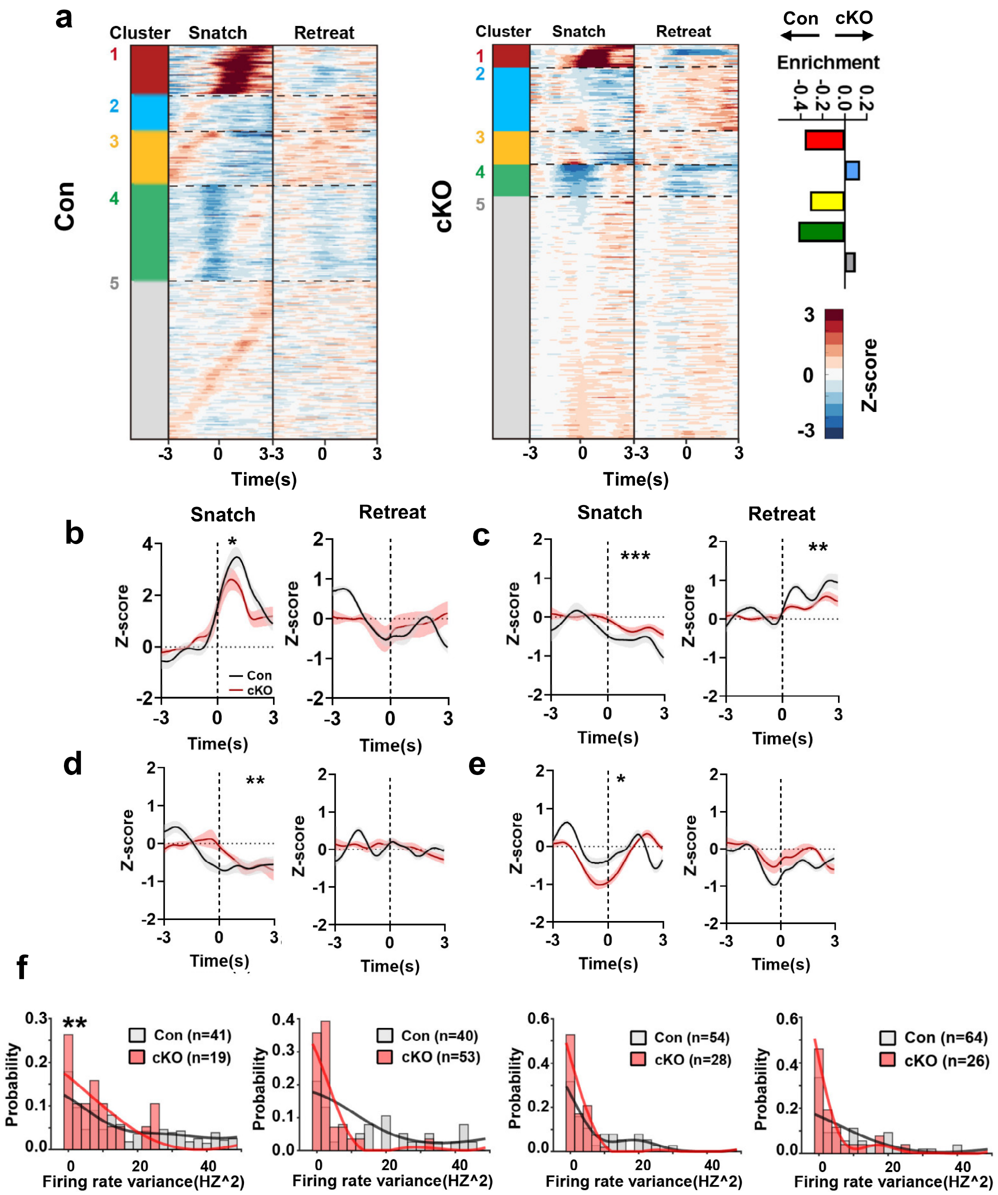

Supplementary Fig. S14| Differences in vHPC neurons responses to specific behaviors between Con/cKO mice during food competition. a Left, heatmap of vHPC neurons responses to two specific behaviors during food competition in Con and cKO mice. Colors represent clusters derived from hierarchical clustering. Cell clusters with a z-score greater than 1.5 or less than -1 were considered responsive to the event (Con n = 199; cKO n = 126). Clusters without responsive are labeled in grey. Right, difference between Con and cKO cells (percentage enrichment) across functional clusters. **b-e** Response magnitude for different cell clusters of vHPC neurons to snatch/retreat behavior events, the dotted line represents the onset of the behavior events. Cluster 1 (b), n(Con) = 41, n(cKO) = 19, snatch $P = 0.0133$, retreat $P = 0.1626$; Cluster 2 (c), n(Con) = 40, n(cKO) = 50, snatch $P < 0.001$, retreat $P < 0.01$; Cluster 3 (d), n(Con) = 54, n(cKO) = 28, snatch $P < 0.01$, retreat $P = 0.8104$; Cluster 4 (e), n(Con) = 64, n(cKO) = 26, snatch $P = 0.0157$, retreat $P = 0.058$; Wilcoxon rank sum test. **f** Distribution of firing rate variance for each neuronal cluster in Con or cKO mice. The line represents the density curve of kernel density estimation using a Gaussian kernel. Black solid line: Con group; Red solid line: cKO group. Cluster 1, $P = 0.0047$; Cluster 2, $P = 0.0815$; Cluster 3, $P = 0.1725$; Cluster 4, $P = 0.5596$; Kolmogorov–Smirnov test.

Supplementary Fig. S15

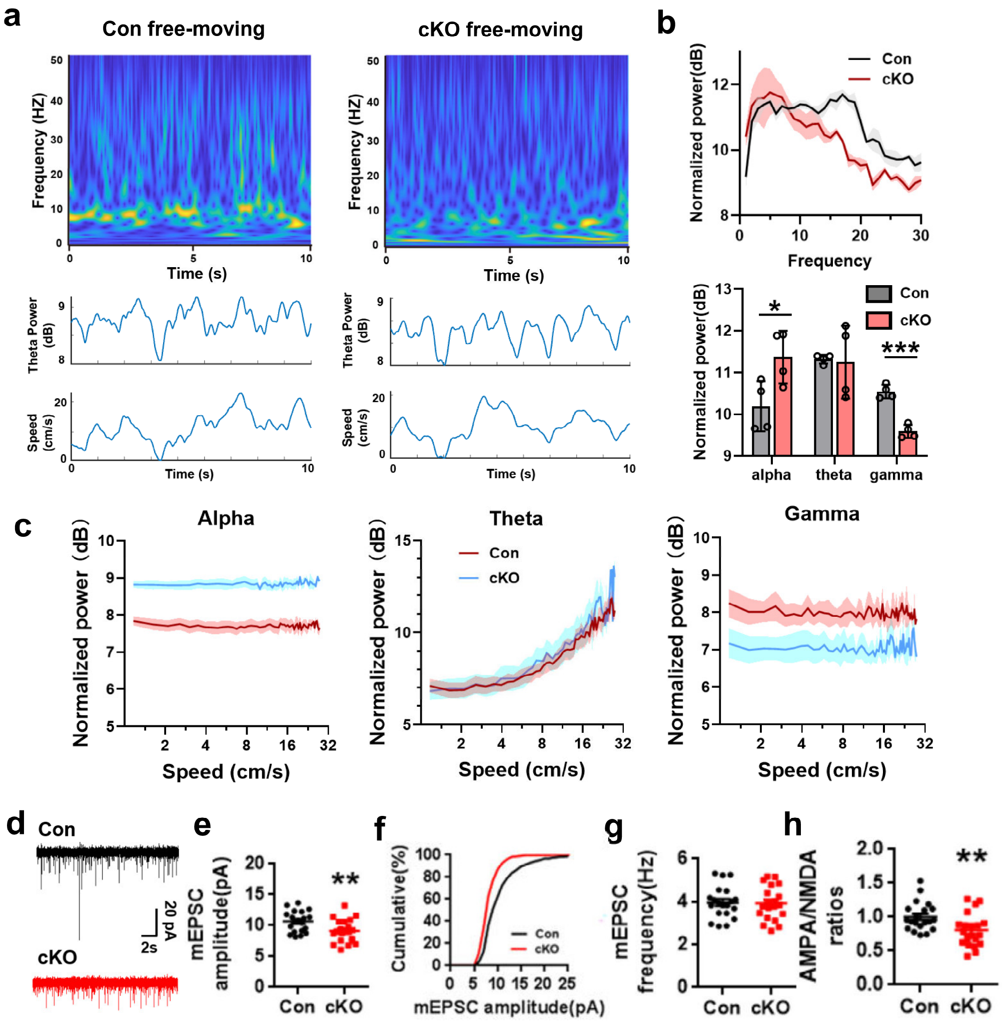

Supplementary Fig. S15| The electrophysiological characteristics in vHPC neurons of *Grina* cKO mice. a (Upper) The examples of spectrograms of vHPC local field potentials (LFP) during free-moving periods. (Bottom) The normalized theta power (dB) and speed (cm/s) of freely moving recorded mice during this segment. Time 0 indicates the start of the segment. **b** (Upper) Power spectra of Con mice or cKO mice during free-moving periods. (Bottom) Energy statistics in different frequency bands of LFP, alpha (0-4 HZ), $P = 0.035$; theta (4-12 HZ), $P = 0.872$; gamma (12-64 HZ), $P < 0.001$; Student’s t test, $n = 4$ mice for each group. **c** The relationship between LFP power of different rhythms and speed in Con or cKO mice ($n = 4$ mice for each group). **d** Representative traces of mEPSCs in hippocampal neurons from cKO mice and control mice. **e-g** The mean mEPSCs amplitudes (e), representative cumulative distribution of mEPSCs amplitudes (f) and frequencies (g). $n = 20$ neurons from five mice of each group. **h** The AMPA/NMDA ratios in Con and cKO mice, respectively. $n = 20$ for each group. The data are presented as means ± SEM, Student’s t test. ** $P < 0.01$.

Supplementary Fig. S16

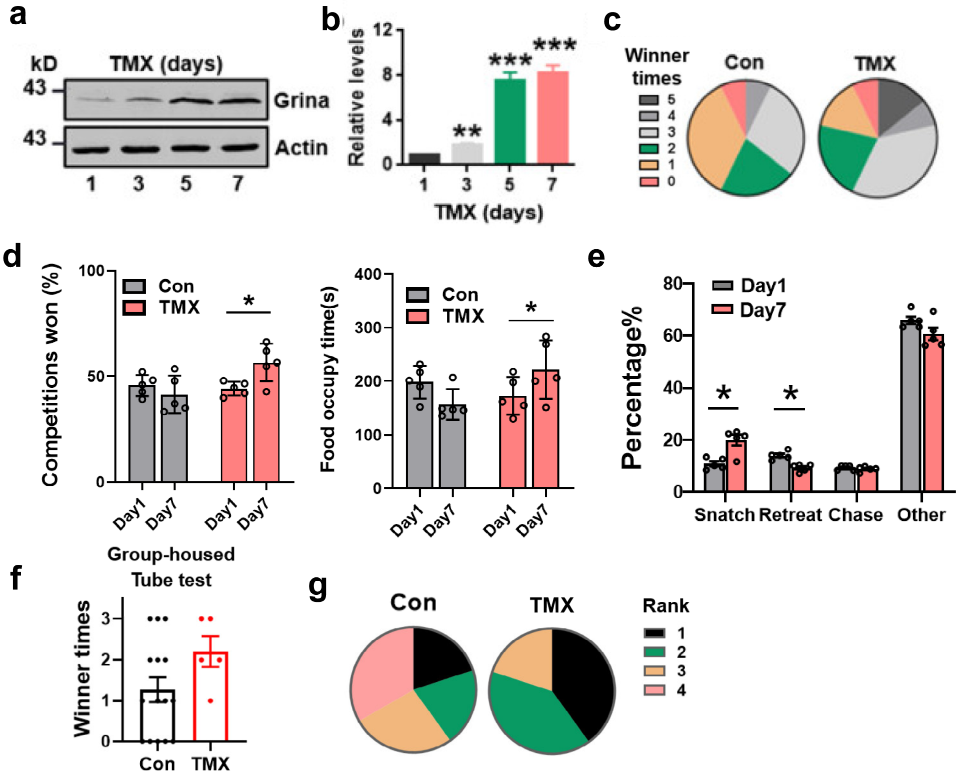

Supplementary Fig. S16| Induction of *Grina* expression restores the behavioral manifestation of cKO mice. a *Grina* protein levels in the ventral hippocampus of virus-injected (AAV2/9-SYN-DIO-Grina-EGFP) HL CreER+/- mice following intraperitoneal administration of tamoxifen (TMX). **b** Quantitative analysis for **a**. Student's t test, $^{**}P < 0.01$, $^{***}P < 0.001$, $n = 3$ for each group. **c** Winner times in the single-housed tube test of Con mice and TMX mice. Chi-square test, x^2 = 8.1, $P < 0.01$. **d** Left: the percentage of the cases that Con or TMX mice won the food competition task on day1 and day 7 (Student's t test, $P < 0.05$, $n = 5$ for each group). Right: total food occupancy time of Con or TMX mice in food competition task on day1 and day 7 (Student's t test, $^{*}P < 0.05$, $n = 5$ for each group). **e** Percentage of frames that correspond to specific behavioral class out of the total frames in TMX round (TMX mice as challengers) at day1 and day 7 (Student's t test, $^{*}P < 0.05$, $n = 5$ for each group) **f** Group-housed tube test, one TMX mice was co-housed with three Con mice for 2 weeks. The winner times in the tube test of each individual mouse in five cages. **g** The rank distribution in the group-housed tube test of Con mice and TMX mice ($n = 20$ mice for five cages). Chi-square test, x^2=10.2, $P < 0.05$.

Supplementary Fig. S17

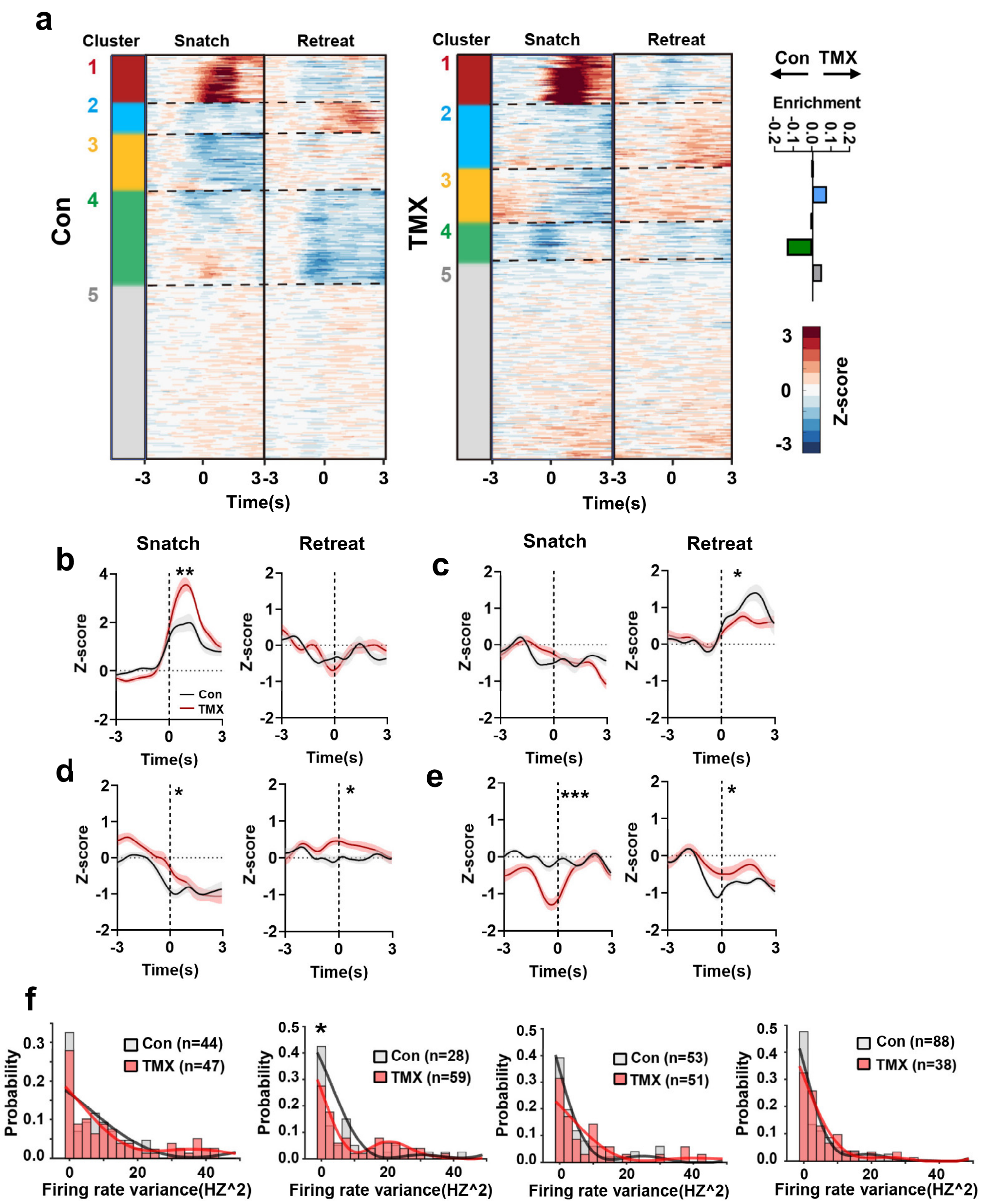

Supplementary Fig. S17| Induction of *Grina* expression restores the neural response in vHPC neurons. a Left, heatmap of vHPC neuron responses to two specific behavioral events in food competition. Cell clusters with a z-score greater than 1.5 or less than -1 were considered responsive to the event (Con n = 218; TMX n = 195). Right, difference between Con and TMX cells (percentage enrichment) across functional clusters. **b-e** Response magnitude for different cell clusters of vHPC neurons to snatch/retreat behavior events. The dotted line represents the onset of the behavior events. Cluster 1 (b), n(Con) = 44, n(TMX) = 47, snatch P = 0.0023, retreat P = 0.0826; Cluster 2 (c), n(Con) = 28, n(TMX) = 59, snatch P = 0.8615, retreat P = 0.0323; Cluster 3 (d), n(Con) = 53, n(TMX) = 51, snatch P = 0.0311, retreat P = 0.0278; Cluster 4 (e), n(Con) = 88, n(TMX) = 38, snatch P < 0.001, retreat P = 0.038; Wilcoxon rank sum test. **f** Distribution of firing rate variance for each neuronal cluster in Con or TMX mice. The line represents the density curve of kernel density estimation using a Gaussian kernel. Black solid line: Con group; Red solid line: TMX group. Cluster 1, P = 0.5596; Cluster 2, P = 0.0425; Cluster 3, P = 0.8186; Cluster 4, P = 0.9780; Kolmogorov–Smirnov test.
