# Supplementary material for: Historical loss weakens competitive behavior by remodeling ventral hippocampal dynamics
Source: Cell Discov. 2025 Feb 25;11:16. doi: 10.1038/s41421-024-00751-3 (PMC11850767; doi:10.1038/s41421-024-00751-3)
Supplement: Supplementary file 3 — Supplementary Table S1 [file 41421_2024_751_MOESM3_ESM.docx]

**Supplementary Video titles**

**Supplementary Video S1. The representative segments of the food competition task.**

**Supplementary Tables**

**Supplementary Table S1. All of the primary antibodies used in this study.**

| **Antibodies** | **Catalog no.** | **Source** | **MAb/RAb** | **Dilution** |
| --- | --- | --- | --- | --- |
| anti-Grina | GTX51232 | GeneTex | R | 1:1000 for WB  1:300 for IHC, IF |
| anti-NeuN | ab104224 | Abcam | M | 1:500 for IF |
| anti-GFAP | ab190288 | Abcam | M | 1: 500 for IF |
| anti-Iba1 | ab15690 | Abcam | M | 1:500 for IF |
| anti-Actin | 60008-1-Ig | Proteintech | M | 1:5000 for WB |
